# Ciliary IFT88 Protects Coordinated Adolescent Growth Plate Ossification From Disruptive Physiological Mechanical Forces

**DOI:** 10.1002/jbmr.4502

**Published:** 2022-02-20

**Authors:** Clarissa R Coveney, Hasmik J Samvelyan, Jadwiga Miotla‐Zarebska, Josephine Carnegie, Emer Chang, C Jonty Corrin, Trystan Coveney, Bryony Stott, Ida Parisi, Claudia Duarte, Tonia L Vincent, Katherine A Staines, Angus KT Wann

**Affiliations:** ^1^ Centre for OA Pathogenesis Versus Arthritis The Kennedy Institute of Rheumatology, University of Oxford Oxford UK; ^2^ School of Pharmacy and Biomolecular Sciences University of Brighton Brighton UK

## Abstract

Compared with our understanding of endochondral ossification, much less is known about the coordinated arrest of growth defined by the narrowing and fusion of the cartilaginous growth plate. Throughout the musculoskeletal system, appropriate cell and tissue responses to mechanical force delineate morphogenesis and ensure lifelong health. It remains unclear how mechanical cues are integrated into many biological programs, including those coordinating the ossification of the adolescent growth plate at the cessation of growth. Primary cilia are microtubule‐based organelles tuning a range of cell activities, including signaling cascades activated or modulated by extracellular biophysical cues. Cilia have been proposed to directly facilitate cell mechanotransduction. To explore the influence of primary cilia in the mouse adolescent limb, we conditionally targeted the ciliary gene Intraflagellar transport protein 88 (*Ift88* ^*fl/fl*^) in the juvenile and adolescent skeleton using a cartilage‐specific, inducible Cre (*AggrecanCre*ER^T2^ *Ift88* ^*fl/fl*^). Deletion of IFT88 in cartilage, which reduced ciliation in the growth plate, disrupted chondrocyte differentiation, cartilage resorption, and mineralization. These effects were largely restricted to peripheral tibial regions beneath the load‐bearing compartments of the knee. These regions were typified by an enlarged population of hypertrophic chondrocytes. Although normal patterns of hedgehog signaling were maintained, targeting IFT88 inhibited hypertrophic chondrocyte VEGF expression and downstream vascular recruitment, osteoclastic activity, and the replacement of cartilage with bone. In control mice, increases to physiological loading also impair ossification in the peripheral growth plate, mimicking the effects of IFT88 deletion. Limb immobilization inhibited changes to VEGF expression and epiphyseal morphology in *Ift88*cKO mice, indicating the effects of depletion of IFT88 in the adolescent growth plate are mechano‐dependent. We propose that during this pivotal phase in adolescent skeletal maturation, ciliary IFT88 protects uniform, coordinated ossification of the growth plate from an otherwise disruptive heterogeneity of physiological mechanical forces. © 2022 The Authors. *Journal of Bone and Mineral Research* published by Wiley Periodicals LLC on behalf of American Society for Bone and Mineral Research (ASBMR).

## Introduction

All biological processes take place in the presence of mechanical forces.^(^ ^1^ ^)^ Biophysical environmental cues must be assimilated into preprogrammed genetic plans; cells and the extracellular matrix (ECM) collectively integrate mechanical forces to orchestrate tissue mechano‐adaptations befitting developmental period and location. The creation, maturation, and homeostasis of the musculoskeletal (MSK) system depends upon the regulated integration of, and balanced response to, mechanical and biological cues. However, how forces are translated into appropriate tissue mechano‐adaptations remains to be understood and is a challenging question to address.

The primary cilium has been proposed to play a central role in cellular mechanotransduction,^(^ ^2^, ^3^, ^4^, ^5^, ^6^ ^)^ but the mechanism by which cilia transduce or influence the cellular response to mechanical force in health and disease is still debated.^(^ ^7^, ^8^, ^9^, ^10^, ^11^ ^)^ A singular, microtubule‐based organelle assembled by the vast majority of cell types, the cilium is a well‐established nexus for the transduction of external cues, acting as a nanoscale scaffold for the regulation of multiple signaling pathways, including growth factor signaling.^(^ ^12^, ^13^, ^14^, ^15^ ^)^ The ciliopathies, congenital disorders associated with mutations to ciliary‐associated genes or biology, have a well‐described MSK subset,^(^ ^16^ ^)^ demonstrating the fundamental importance of the primary cilium in human skeletal development. The developmental depletion of key ciliary genes in the mouse^(^ ^17^, ^18^, ^19^, ^20^, ^21^, ^22^, ^23^ ^)^ results in impaired growth and premature epiphyseal fusion when the growth plate (GP), the cartilaginous template for long bone formation, fuses to become bone early. Far less is known about ciliary influence in adulthood, but ciliary IFT88 remains influential in postnatal articular cartilage.^(^ ^24^ ^)^

Longitudinal bone growth is underpinned by endochondral ossification (EO), a carefully coordinated process of cell and tissue differentiation, that ultimately results in GP cartilage being replaced by bone. Elongation requires the GP to be organized into columns of chondrocytes, continuously supplied, throughout growth, by a stem cell niche.^(^ ^25^, ^26^ ^)^ Progeny of this niche undergo proliferation, enlargement through hypertrophic differentiation,^(^ ^27^ ^)^ and ultimately either apoptosis or transdifferentiation,^(^ ^28^ ^)^ all the while secreting and remodeling a regionally specialized extracellular matrix. Thus, a highly organized sequence of cellular and extracellular signaling events enables dynamic, almost simultaneous mineralization and resorption of cartilage, vascular invasion, and the creation of bone. During EO, complex gradients of growth factor signaling coordinate differentiation of cells and matrix. One example of signaling underpinning this program of differentiation is the Indian hedgehog (Ihh)‐parathyroid hormone‐related protein (PThrP) feedback loop, which acts to balance proliferation and hypertrophic differentiation.^(^ ^29^, ^30^, ^31^, ^32^, ^33^ ^)^ In a similar fashion to targeting cilia in early development, as cilia are central regulators of Hedgehog (Hh) signaling, disruption of this loop by genetic perturbation results in accelerated GP closure.^(^ ^12^, ^13^, ^15^ ^)^ Comparatively to EO, the signaling events underlying the abrupt discontinuation of EO, demarcating the cessation of growth, are poorly understood.

Both Hh signaling, PThrP signaling, and their downstream effects have themselves been previously demonstrated to be mechano‐regulated. For example, hydrostatic strain applied to GP chondrocytes results in increased Ihh signaling and proliferation.^(^ ^34^ ^)^ Indeed, either by modulation of the expression of ligands, receptors, or by their release from sequestration within the matrix, growth factor signaling in cartilage and bone is highly mechano‐regulated.^(^ ^35^, ^36^, ^37^ ^)^ A number of studies illustrate the importance of mechanics in animal models of bone growth. In the absence of mechanical forces exerted by muscular contraction, proliferation decreased in the GP of embryonic chicks.^(^ ^38^, ^39^ ^)^ Tissue mechanics are also required for the intercalation of growth plate chondrocytes to affect extension.^(^ ^40^, ^41^ ^)^ Despite the importance of mechanotransduction to skeletal development, health, and disease, the cellular and molecular components that might comprise a system that supports mechanical homeostasis in cartilage and many other tissues, analogous to the bone mechanostat originally proposed by Frost,^(^ ^42^ ^)^ remain elusive.

We hypothesized that IFT88, and by extension the primary cilium, would maintain profound influence in the postnatal growth plate. To test this, we used a conditional and inducible Cre approach (*AggrecanCreER* ^*T2*^;*Ift88* ^*fl/fl*^). We show ciliary IFT88 plays an instrumental role in coordinating adolescent epiphyseal biology in vivo. In light of our results, we propose that cilia protect the carefully orchestrated cessation of growth defined by terminal mineralization of the GP from otherwise disruptive mechanical forces. Furthermore, we offer a new paradigm for the role of cilia in tissue mechanotransduction during morphogenesis.

## Materials and Methods

### Animals

All mice were housed in the biomedical services unit (BSU) at the Kennedy Institute, within the University of Oxford. Mice were housed 4 to 7 per standard, individually ventilated cages and maintained under 12‐hour light/dark conditions at an ambient temperature of 21°C. *Ift88* ^*fl/fl*^ mice were obtained from Jackson Laboratory (Bar Harbor, ME, USA; stock no. 022409) and maintained as the control line, and in parallel offspring were crossed with the *AggrecanCreER* ^*T2*^ mouse line, *AggrecanCreER* ^*T2*^;*Ift88* ^*fl/fl*^ (*Ift88* cKO), originally generated at the Kennedy Institute of Rheumatology.^(^ ^43^ ^)^ The TdTomato reporter mouse line *B6.Cg‐Gt(ROSA)26Sor* ^*tm14(CAG‐TdTomato)Hze*^ */J* was originally from Jackson Laboratory (stock no. 007914). For all experiments, apart from double neurectomy (off‐loaded) and wheel exercised (male only), both sexes were used and no effect of sex was observed in the data. Animal husbandry and experiments were conducted in accordance with University of Oxford ethical frameworks and under UK Personal (Coveney) and Project (Vincent) licenses as granted by the UK Home Office.

### Antibodies

The following primary antibodies were used for immunohistochemistry (IHC) in tandem with Invitrogen (Carlsbad, CA, USA) AlexaFluor secondaries: acetylated‐a‐tubulin (6‐11B‐1, MilliporeSigma, Burlington, MA, USA), Arl13b (Proteintech, Rosemont, IL, USA; 17711‐1‐AP). Anti‐type X collagen (polyclonal, Abcam, Cambridge, MA, USA; ab58632), Anti‐CD31 (goat IgG, R&D Systems, Minneapolis, MN, USA; AF3628) Anti‐Vegf (monoclonal, Abcam, ab232858).

### Tamoxifen treatment

Tamoxifen (Sigma‐Aldrich, St. Louis, MO, USA; catalog no. T5648) was dissolved in 90% sunflower oil and 10% ethanol at a concentration of 20 mg/mL by sonication. Tamoxifen was administered via intraperitoneal injection at ages according to experimental requirement, on 3 consecutive days at 50 to 100 mg/kg (dependent on animal weight). In naïve animals, these injections began at 4, 6, or 8 weeks of age.

### Double neurectomy

For double neurectomy experiments, tamoxifen was administered in the days before surgery. One or 2 days before surgery, mice were transferred to cages containing soft bedding. After surgery, animals still have full access to food and water and move freely, albeit dragging the immobilized limb and therefore likely placing more weight on the contralateral. Briefly, animals were prepared for surgery, anesthesia, and analgesia as previously described,^(^ ^44^ ^)^ and the right hind limb was shaved from the knee up to the hip and in the groin. Fur on the back just above the right limb is also shaved to expose the area from the spine to the flank on the right‐hand side. Using a 3 mm size 15 ophthalmic scalpel (MSP, Aguadilla, Puerto Rico), a longitudinal incision is made from the right knee joint up and inward toward the groin. Fine‐toothed forceps were used to separate the overlying skin to reveal the muscle, femoral artery, and the femoral nerve running in very close proximity. Using curved forceps, the femoral nerve is separated from its soft tissue attachments underneath. The femoral nerve is carefully transected using micro‐dissecting scissors and a 0.5 cm section is removed. The wound was closed using the above suturing method with additional sutures added as required. The mouse is turned onto its front and the right hind limb is stretched out. Using a 3 mm size 15 ophthalmic scalpel (MSP), an incision of approximately 2 cm is made from the spine outward. Using curved forceps, the overlying skin is separated to reveal the muscle and the sciatic nerve. The curved forceps are inserted under the sciatic nerve to separate it from the surrounding tissue. A 2 to 4 mm region of the sciatic nerve is removed. After this, the wound was sutured using the described method above with additional sutures added as required. Mice were transferred to a recovery chamber as described above and recovered within 10 minutes of anesthetic withdrawal. Mice were subsequently housed in soft bedding without environment‐enhancing balconies or tubes to prevent aggravation of exposed skin. Animals were disturbed as little as possible. Sudocrem was applied to the skin of the foot if aggravated. For wheel‐exercise mouse experiments, animals had access to a wheel for 2 weeks after tamoxifen at 8 weeks of age.

### Micro‐CT BV/TV

Knee joints were imaged using a micro‐CT scanner (SkyScan 1172 X‐ray microtomograph, Bruker microCT, Kontich, Belgium) within 70% ethanol (10 μm/pixel, 3 minutes of acquisition time).^(^ ^45^ ^)^ Using the CTan (Bruker) program, saved image sequences were opened in the software to conduct 3D parameter analysis. Regions of interest including the epiphysis and the bone directly underneath the epiphyseal plate were defined and used to calculate the bone volume (BV), total volume (TV), and ratio of BV to TV (BV/TV).

### Bridging analysis

Scans were performed with an 1172 X‐Ray microtomograph (SkyScan, Bruker). The high‐resolution scans with a pixel size of 5 μm were imaged. The applied X‐ray voltage was 50 kV, X‐ray intensity 200 μA with a 0.5 mm aluminum filtration. The scans were taken over 180 degrees with a 0.7‐degree rotation step. The images were reconstructed and binarized with a threshold of 0 to 0.16, and ring artifact reduction was set at 10 using the SkyScan NRecon software package (v. 1.6.9.4, SkyScan, Bruker). The images then were realigned vertically using DataViewer software (v. 1.5.1.2 64‐bit, SkyScan, Bruker) to ensure similar orientation for bridging analysis. Bony bridging was analyzed using a 3D quantification method as previously described.^(46^ ^)^ Micro‐CT scans of the tibias were segmented using Avizo software (v. 8.0, VSG, Burlington, VT, USA). The volume images were manually aligned along with the metaphyseal tibial shaft and central point of each individual bridge was selected, quantified, and projected onto the tibial joint surface. From this, the areal number density of bridges (*N*, per 256 μm × 256 μm window) was then calculated, and the distribution was superimposed on the tibial surface (each bridge has a color that represents the areal number density at the bridge location).

### Growth plate cartilage measurements

Images of histology were taken using an Olympus Osteometric microscope using a 10× lens. Quantification of cartilage width was conducted with Image J (NIH, Bethesda, MD, USA). To assess growth plate length from the lateral, medial, and middle regions, maximum measurements were taken from three consecutive sections from the middle of the joint (nine measurements per mouse). To find the length of non‐hypertrophic region, the length of the hypertrophic region was taken away from growth plate length.

### Histology

Knee joints were harvested into 10% neutral‐buffered formalin (CellPath, Newtown, UK) for 24 to 48 hours. Joints were decalcified (EDTA), paraffin embedded, and coronally sectioned through the entire depth of the joint. Sections (4 μm) at 80 μm intervals were stained with Safranin O.

### TRAP staining

An amount of 70 mg Napthol AS‐TR phosphate disodium salt (Sigma) was dissolved in 250 μL NN‐dimethyl formamide (Sigma) and added to 50 mL of 0.2 M sodium acetate buffer at pH 5.2. 2. An amount of 115 mg sodium tartrate dihydrate (Sigma) and 70 mg of fast red salt TR 1,5‐napthalenedisulfonate (Sigma) was dissolved into this solution. Fixed, decalcified, unstained coronal knee sections were deparaffinized, rehydrated, and placed into this solution and incubated at 37°C for 2 hours. Sections were washed briefly in deionized water and counterstained with Meyer's hemotoxylin (Sigma) for 1 minute and washed in deionized water before being mounted in aqueous mounting medium.

### Von Kossa staining

Cryosections (164 μm) of knee joints were defrosted in deionized water for 5 minutes and incubated for 7 minutes under UV light in 5% aqueous silver nitrate. Sections were rinsed thoroughly in deionized water and placed in sodium thiosulfate for 5 minutes, rinsed, and then counterstained with Neutral red (1%) solution for 2 minutes. Slides were dehydrated and mounted in Prolong Gold and visualized.

### TUNEL

In situ detection of apoptosis was conducted using TACS 2 Tdt‐Fluor In Situ Apoptosis Kit (Trevigen, Gaithersburg, MD, USA; 4812‐30‐K) after deparaffinizing sections. Because the phenotypes were so apparent both on micro‐CT and by histology, we did not implement blinded analyses.

### Immunohistochemistry

Fixed, decalcified, unstained coronal knee sections were deparaffinized, rehydrated, quenched in 0.3 M glycine, and treated with proteinase K for 30 minutes. Samples underwent chondroitinase (0.1 U) treatment for 30 minutes at 37°C, permeabilized by 0.2% Triton X‐100 for 15 minutes, and blocked in 5% goat serum and 10% bovine serum albumin (BSA) in phosphate‐buffered saline. Samples were incubated with primary antibody or IgG control, or no primary, overnight at 4°C. Sections were washed and incubated with Alexa‐conjugated 555 secondary antibodies for 30 minutes. Samples were incubated with nuclear stain DAPI (1:5000), before mounting in Prolong Gold and visualized.

### Cilia staining and confocal

Knee joints were harvested in ice‐cold 4% PFA and incubated in the fridge for 24 hours. Knee joints were subsequently transferred to ice‐cold 10% sucrose for 24 hours. This was repeated with 20% and 30% ice‐cold sucrose. Knee joints were then embedded into Super Cryo Embedding Medium (C‐EM001, Section‐lab Co. Ltd, Hiroshima, Japan) and stored at −80°C before 16 μm sections were collected using a precooled cryotome at −16°C with Cryofilm type 3C (16UF) 2.5 cm C‐FUF304. Sections were stored at −80°C. Slides were hydrated for 5 minutes in 1× phosphate‐buffered saline (PBS) and fixed for 10 minutes with 4% formaldehyde 0.2% Triton X‐100 in PBS. Sections were incubated in blocking buffer (10% bovine serum albumin, 5% goat serum in PBS) for 10 minutes, followed by a 45‐minute incubation at room temperature and pressure (RTP) with primary antibody diluted in blocking buffer (1:1000 ac‐a‐tubulin, 1:500 Arl13b). After three 5‐minute washes in PBS, sections were incubated with Alexa‐conjugated 555 secondary antibodies for 30 minutes diluted in blocking buffer (1:500). After three 5‐minute washes in PBS, nuclei were stained using 1:5000 DAPI diluted in PBS for 5 minutes, washed once in PBS, and mounted in prolong gold. Imaging and analysis images were acquired using an Olympus FluoView FV1000 Confocal Microscope (Olympus, Tokyo, Japan) with an oil immersion 63× objective to produce confocal serial sections for maximum‐intensity z‐stack (4.6 to 5.2 μm thick) reconstruction of GP sections with laser voltage, offset and gain held constant. Six images of the growth plate per joint across the width of the tibia were taken and reconstructed. Cilia‐positive and cilia‐negative cells were blind‐counted by two individuals and their analysis averaged for each joint.

### RNAscope

Knee joints were harvested into ice‐cold 4% PFA and incubated in the fridge for 24 hours. Knee joints were subsequently transferred to ice‐cold 10% sucrose for 24 hours. This was repeated with 20% and 30% ice‐cold sucrose. Knee joints were then embedded into Super Cryo Embedding Medium (C‐EM001, Section‐lab Co. Ltd) and stored at −80°C before 8 μm sections were collected using a precooled cryotome at −16°C with Cryofilm type 3C (16UF) 2.5 cm C‐FUF304. Sections were stored at −80°C. Slides were washed with PBS for 5 minutes and then baked at 60°C for 30 minutes. Slides were fixed using ice‐cold 4% PFA for 15 minutes at 4°C. Increasing concentrations of ethanol made in milli‐Q water was applied, 50%, 70%, 100%, and 100% fresh ethanol, 5 minutes for each gradient. The sample was air‐dried for 5 minutes and incubated with hydrogen peroxide (PN 322381) for 10 minutes. Slides were submerged twice in milli‐Q water and then transferred into prewarmed Target Retrieval Buffer (1×) (322000) in the steamer for 10 minutes at 75°C. Slides were washed briefly in milli‐Q water before being submerged briefly in 100% ethanol and air‐dried for 5 minutes. Protease III (PN 322381) was used to cover the sample and incubated in a HybEZ Oven at 40°C for 30 minutes. Slides were submerged briefly in milli‐Q water. RNAscope Multiplex Fluorescent Reagent Kit v2 Assay reagents (323100) was subsequently followed. We used RNAscope Probe‐Mm‐Gli1‐C2 (311001‐C2) to assess Gli1 expression in GP cartilage and Opal 690 Reagent Pack FP1497001KT for visualization. Lateral, middle, and medial regions of GP were images using a 60× lens, 520 nm/px, 377.6 μm × 619.77 μm, using a Zeiss 980 (Zeiss Microscopy, Jena, Germany) confocal microscope. After normalization with positive (RNAscope 3‐plex Positive Control Probe‐Mm, PPIB gene, 320881) and negative (RNAscope 3‐plex Negative Control Probe‐Mm, bacterial dapB gene, 320871) control probes, the number of Gli1‐positive and ‐negative nuclei were counted and averaged across the three regions per mouse.

## Results

### Deletion of IFT88 in the juvenile and adolescent growth plate inhibits endochondral ossification and growth plate narrowing

**Fig. 1:**
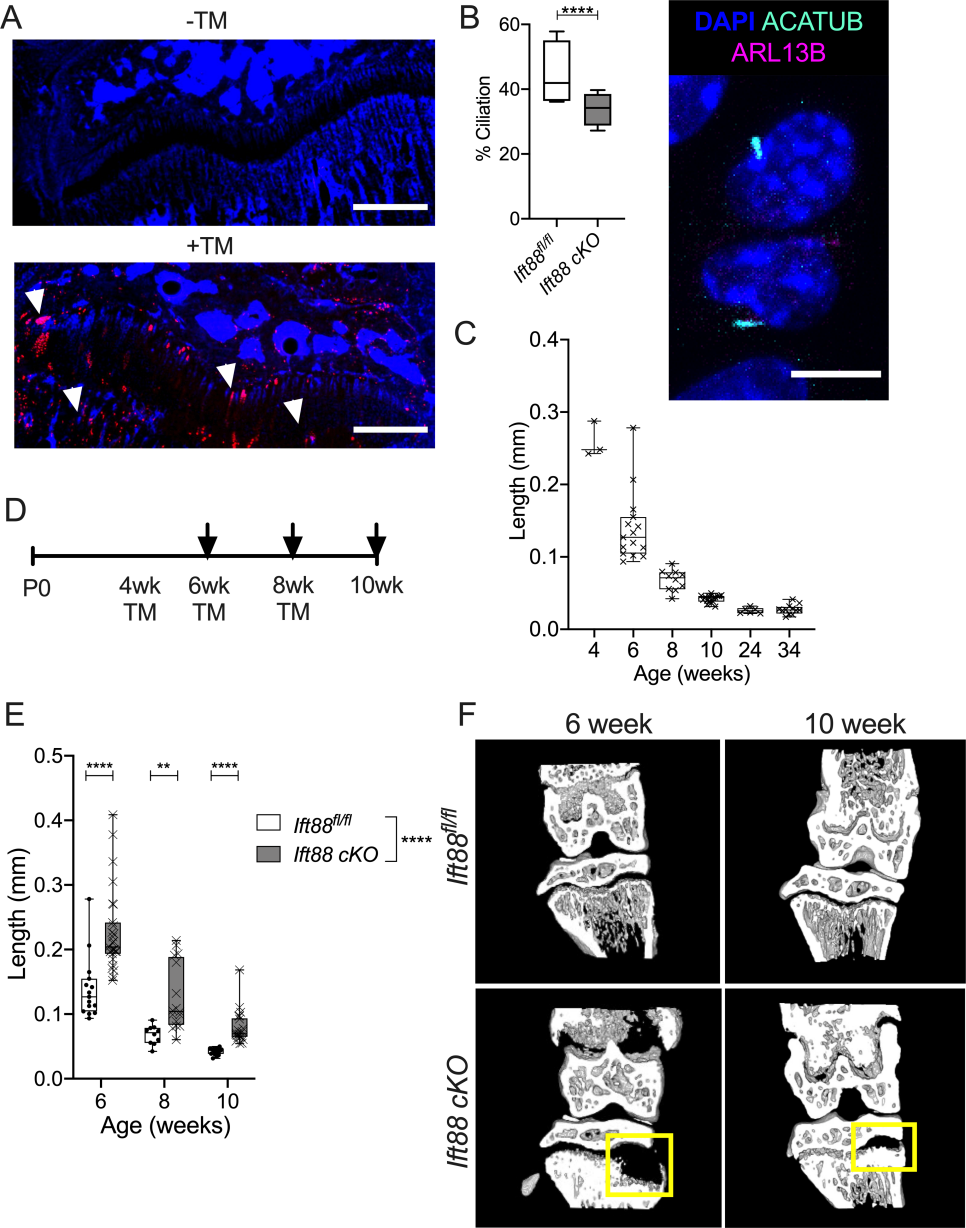
Deletion of IFT88 reduces growth plate ciliation and inhibits growth plate narrowing. (*A*) Cryosections of knee joints counterstained with nuclear DAPI were taken from 6‐week‐old AggrecanCreER^T2^;TdTomato mice that received tamoxifen (TM) at 4 weeks of age (scale bar = 500 μm). White arrows point to chondrocyte populations exhibiting TdTomato reporter activity. (*B*) Box plot (bars, maximum and minimum values; box is upper quartile and lower quartile with median) depicts percentage ciliation in 10‐week‐old GP in AggrecanCreER^T2^;Ift88^fl/fl^ and Ift88^fl/fl^ control mice 2 weeks after tamoxifen. ****p = <0.0001, Fisher's exact test, contingency data shown in Supplemental Fig. S1 *B*. Image depicts primary cilia staining in GP chondrocytes in situ. (*C*) Box plot depicts GP length of control animals (also treated with tamoxifen) at 4, 6, 8, 10, 24, and 34 weeks of age. *n* = 3 at 4 weeks, 6–17 at all other time points. (*D*) Schematic indicates age of tamoxifen administration (TM) and collection (arrows). (*E*) Box plot depicts GP lengths of control (Ift88^fl/fl^) and AggrecanCreER^T2^;Ift88^fl/fl^ (cKO) mice at 6, 8, and 10 weeks of age as measured from micro‐CT images. *n* = 10–27. (*F*) Partial 3D construction of micro‐CT scans at 6 and 10 weeks of age. Points represent mean GP length per animal, lines at median and interquartile range in violin plots. Genotype effect analyzed by two‐way ANOVA, pairwise analysis by unpaired *t* tests corrected for multiplicity and using a 1% false discovery rate (FDR), *p < 0.05, ****p < 0.0001.

To delete *Ift88*, in a cartilage‐specific, inducible manner, *AggrecanCreER* ^*T2*^;*Ift88* ^*fl/fl*^ (*Ift88* cKO) mice were generated. First, to assess efficacy of Cre recombination in GP chondrocytes, the *AggrecanCreER* ^*T2*^ was crossed with a TdTomato reporter line. Effective Cre recombination was identified in many, but not all, GP chondrocyte columns after tamoxifen administration (*n* = 3) (Fig. 1*A*). Chondrocyte columns expressing Tdtomato were evenly spread throughout the GP, with no bias to particular regions. By the point of analysis, 2 weeks post‐tamoxifen, cells within the primary spongiosa also expressed Tdtomato. IHC staining of cryosections from 10‐week‐old mice enabled visualization of cilia in situ (Fig. 1 *B*, Supplemental Fig. S1 *A*) in control (*Ift88* ^*fl/fl*^) and *AggrecanCreER* ^*T2*^;*Ift88* ^*fl/fl*^ (*Ift88* cKO) mice. Analysis of this staining indicated a ~20% reduction in cilia prevalence in GP chondrocytes (Fig. 1*B*, ****p < 0.0001, Fisher's exact test, contingency data shown in Supplemental Fig. S1 *B*, *n* = 4 in each group). While in the mouse the GP never fully fuses, on approach to skeletal maturity, the rate of longitudinal bone growth decreases between 4 and 10 weeks of age, and tibial GP length reduces with age from approximately 0.26 mm to 0.04 mm (Fig. 1*C*), indicative of GP closure. Tamoxifen was administered to control and *AggrecanCreER* ^*T2*^;*Ift88* ^*fl/fl*^ mice (*Ift88* cKO) at 4, 6, or 8 weeks of age (Fig. 1*D*). GP lengths were analyzed 2 weeks later, using micro‐CT images of whole knee joints, taking the mean of eight length measurements across the full width (Supplemental Fig. S1 *C*). Analysis revealed deletion of IFT88 resulted in statistically significantly longer GP at all time points when compared with controls (****p < 0.0001, two‐way ANOVA, Fig. 1*E*). While variance increased, GP length in *AggrecanCreER* ^*T2*^;*Ift88* ^*fl/fl*^ remained similar to that of control mice at the age tamoxifen was administered (2 weeks prior). Thus, GP narrowing during each these 2‐week periods was effectively abolished. The increases in GP length were also evident in Safranin O‐stained histological sections (GP length quantified in Supplemental Fig. S1 *D*).

Although not the focus of this study, it was clear that the bone in the tibial diaphysis, particularly at 6 weeks of age, appeared increased in density. Analysis of the region of bone directly beneath the GP revealed an increase in BV/TV at 6 weeks of age (Supplemental Fig. S2*A*). Strikingly, elongated cartilaginous GP in *AggrecanCreER* ^*T2*^;*Ift88* ^*fl/fl*^ were characterized by large regions with little or no mineral density that were largely restricted to one or both sides of the tibia (Fig. 1*F*).

### IFT88 deletion preferentially disrupts ossification of the peripheral growth plate

**Fig. 2:**
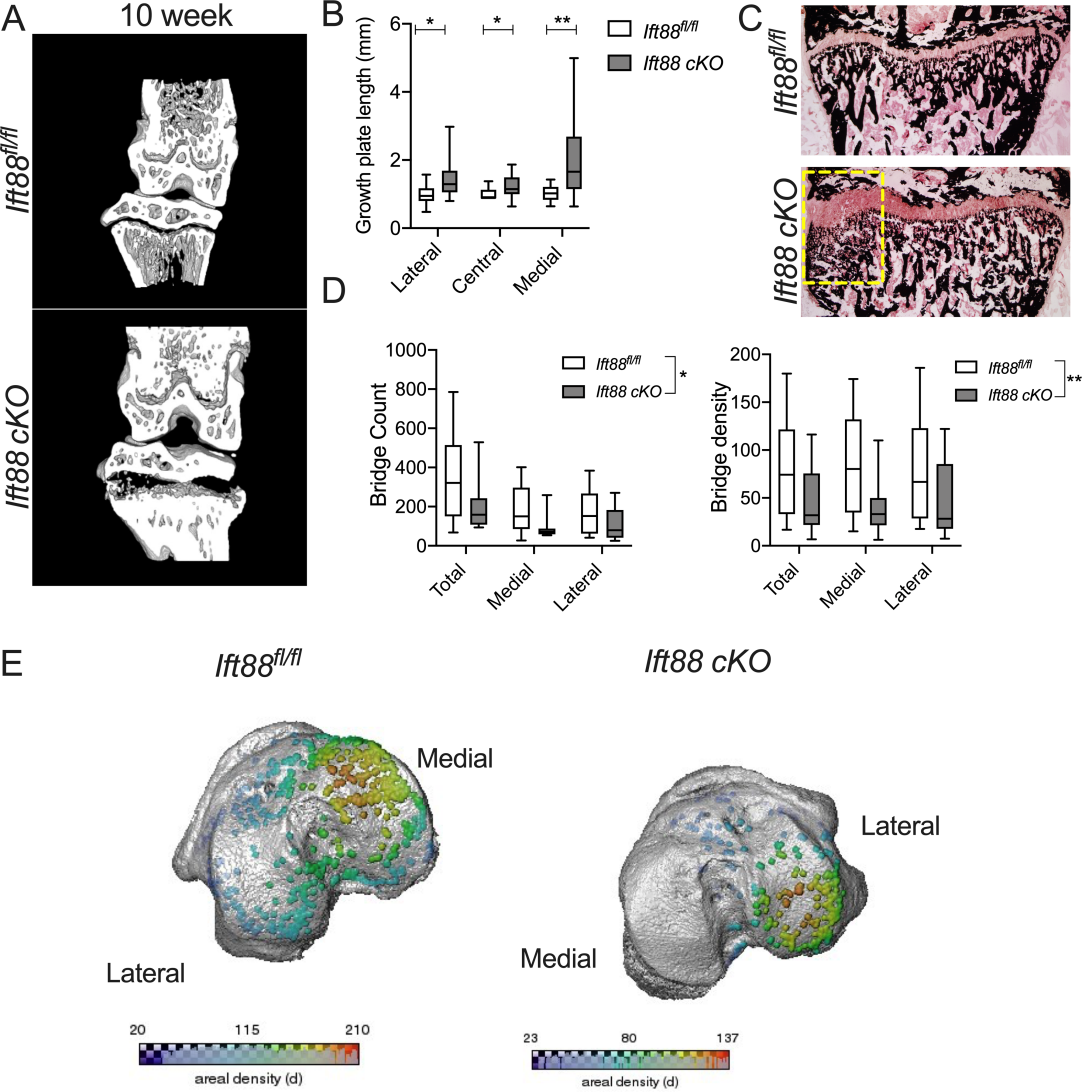
IFT88 deletion inhibits peripheral growth plate ossification. (*A*) Micro‐CT partial 3D construction of AggrecanCreER^T2^;Ift88^fl/fl^ mice at 10 weeks of age. (*B*) Box plots depict (bars, maximum and minimum values; box is upper quartile and lower quartile with median) maximum growth plate lengths taken from the lateral, medial, and central sections of the GP of AggrecanCreER^T2^;Ift88^fl/fl^ animals, normalized to the Ift88^fl/fl^. Analysis by pairwise unpaired *t* tests corrected for multiplicity, FDR 1%, *p = <0.05, **p = <0.01 Ift88^fl/fl^ *n* = 17; AggrecanCreER^T2^;Ift88^fl/fl^ *n* = 19. (*C*) Von Kossa staining of Ift88^fl/fl^ and AggrecanCreER^T2^;Ift88^fl/fl^. Label highlights region of medial bone with disorganized trabeculae. (*D*) Box plots depict number of bridges (count) and bridge density in control and AggrecanCreER^T2^;Ift88^fl/fl^. Points represent median value per animal. Analyzed by two‐way ANOVA, *p < 0.05, **p < 0.01, Ift88^fl/fl^ *n* = 8; AggrecanCreER^T2^;Ift88^fl/fl^ *n* = 8. (*E*) 3D representation mapping GP bridges across tibial articular surfaces of the knee. Color scale indicates the density of the bridges.

Micro‐CT images suggested the effects of IFT88 deletion were restricted to the peripheral regions of the GP, directly below the load‐bearing articular surfaces of the knee (Fig. 2*A*), whereas comparatively the central region of the GP had narrowed normally through ossification. Considering only the 8‐ to 10‐week period, to focus on GP fusion processes and avoid the confounding effect of tibial widening at earlier time points (Supplemental Fig. S2*B*), maximum GP length measurements were taken in lateral, central, and medial regions of control and *AggrecanCreER* ^*T2*^;*Ift88* ^*fl/fl*^ mice and plotted relative to control animals (Fig. 2*B*). This analysis revealed the largest effects were observed in the medial peripheral region of the GP, where GP length was twice that of controls. Comparatively modest effects on GP length were measurable in the lateral region, whereas only very small, but nevertheless statistically significant, differences were observed centrally (Fig. 2*B*). These trends were supported by GP length analysis of histological sections (Supplemental Fig. S2*C*, *D*). Von Kossa staining also indicated disruption to mineralization and trabecular organization beneath the peripheral regions of failed ossification in *AggrecanCreER* ^*T2*^;*Ift88* ^*fl/fl*^ mice (Fig. 2*C*). Previous studies investigating mouse GP ossification describe bone bridging events associated with heterogenous local tissue mechanical stresses.^(^ ^46^ ^)^ Fewer and lower‐density bone bridges were observed in *AggrecanCreER* ^*T2*^;*Ift88* ^*fl/fl*^ mice compared with controls (Fig. 2*D*). This reduction in bridging was again particularly striking on the medial side of the limb (Fig. 2*E*).

### Increased physiological loading disrupts peripheral growth plate

**Fig. 3:**
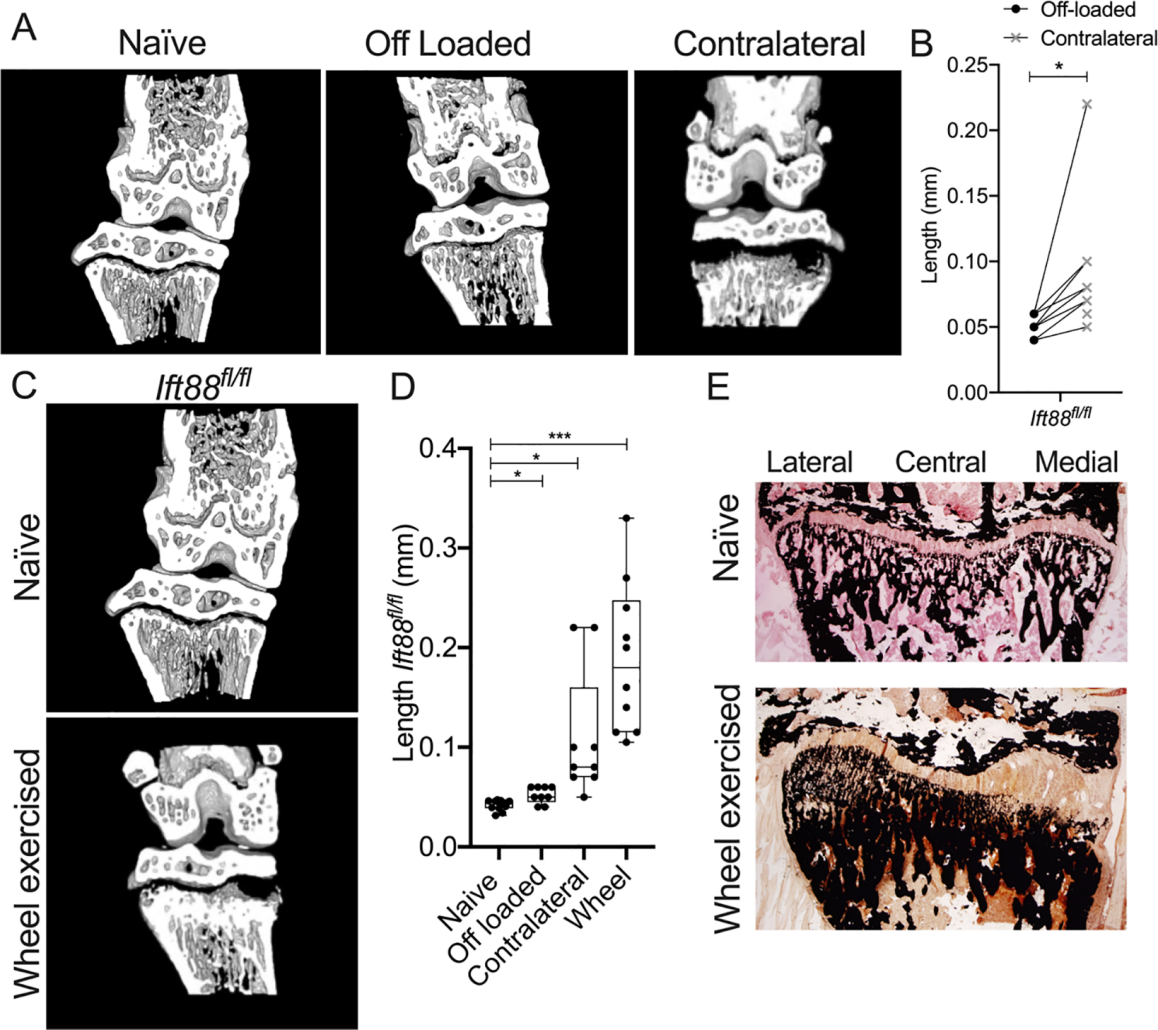
Acute increases in physiological limb loading inhibit peripheral growth plate dynamics. (*A*) Micro‐CT partial 3D construction of off‐loaded and contralateral joints from Ift88^fl/fl^ control mice. (*B*) GP lengths of paired off‐loaded (right) and contralateral (left) joints in Ift88^fl/fl^ control mice. *n* = 8. (*C*) Micro‐CT partial 3D construction of joints from Ift88^fl/fl^ control mice in naïve and after 2 weeks of wheel exercise. (*D*) Box plot (bars, maximum and minimum values; box is upper quartile and lower quartile with median) depicts quantitation of GP length of naïve, off‐loaded, contralateral, and wheel‐exercised Ift88^fl/fl^ control mice. Analyzed by one‐way ANOVA, *p < 0.05, ****p < 0.0001, *n* = 9–12. (*E*) Von Kossa staining of Ift88^fl/fl^ in naïve and after 2 weeks of wheel exercise, both at 10 weeks of age.

Previous modeling has indicated heterogeneity of physiological mechanical stresses across the width of the tibial GP during limb loading^(^ ^46^, ^47^ ^)^ with greatest stresses at the periphery. Given the peripheral, preferentially medial pattern to the failed ossification in *AggrecanCreER* ^*T2*^;*Ift88* ^*fl/fl*^ mice, we hypothesized that coordinated epiphyseal ossification is sensitive to the depletion of IFT88, due to a critical role for cilia in mechanosensation/transduction, as has been previously proposed.^(^ ^48^, ^49^ ^)^ Therefore, we surmised GP narrowing in control mice, between 8 and 10 weeks of age, would be sensitive to acute changes in limb loading. First, we tested the effect of removing mechanical input to the adolescent GP, hypothesizing this may inhibit GP dynamics in a similar manner to that observed upon depletion of cilia. Double neurectomy was performed on the right hind limb at 8 weeks of age. Cutting both the femoral and sciatic nerves rendered the right hind limb incapable of weight bearing (off‐loaded), whereas the left (contralateral) became the predominant weight‐bearing hind limb, taking increased load by means of compensation. Micro‐CT revealed GP in off‐loaded limbs of 10‐week‐old *Ift88* ^*fl/fl*^ control mice were not strikingly different when compared with naïve *Ift88* ^*fl/fl*^ control mice (Fig. 3*A*). Quantification (Fig. 3*D*) did reveal a small but statistically significant increase in GP length with off‐loading, but relative narrowing, across the width of the limb, was uniform. However, the contralateral limbs of operated animals exhibited similar bilateral regions of failed ossification to that observed in naïve *AggrecanCreER* ^*T2*^;*Ift88* ^*fl/fl*^ mice (Fig. 3*A*, right‐hand image), with an associated increase in average GP length when compared with paired off‐loaded limbs (Fig. 3*B*). To further investigate whether increases, rather than decreases, to physiological loading disrupt coordinated GP ossification, mice were given access to free wheel exercise between 8 and 10 weeks of age. At 10 weeks of age, exercised *Ift88* ^*fl/fl*^ control mice also exhibited inhibition of GP ossification in the periphery, again often most pronounced on the medial side (Fig. 3*C*, relative quantification of mean GP length in Fig. 3*D*). Von Kossa staining confirmed a failure of mineralization and alterations to bone architecture beneath in control mice after 2 weeks of wheel exercise (Fig. 3*E*). Von Kossa indicated the inhibitory effects on mineralization were greatest on the medial side but that the mineralized architecture beneath the lateral plateaus was also altered.

### Limb immobilization restores normal growth plate ossification in IFT88cKO mice

**Fig. 4:**
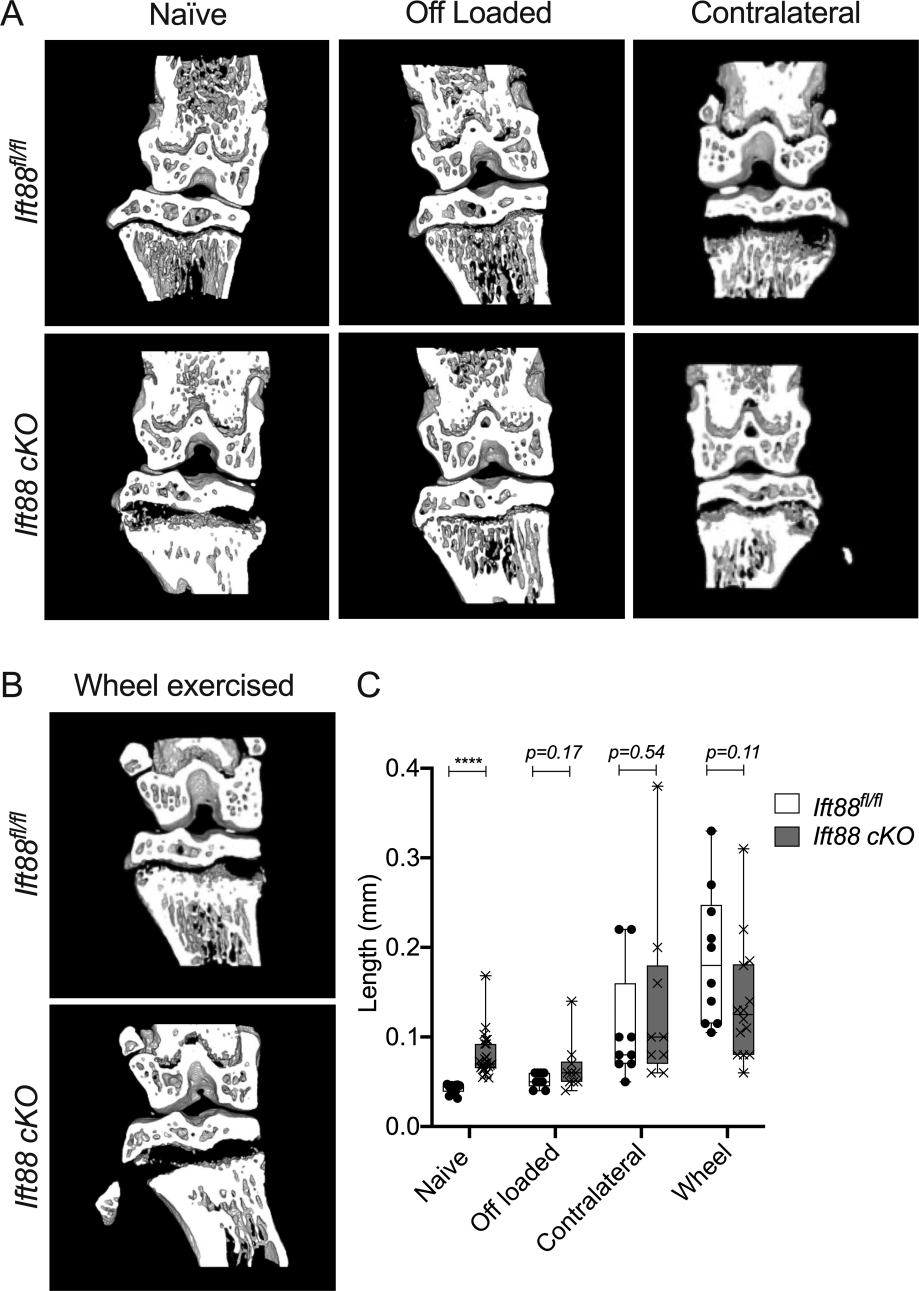
Limb immobilization inhibits the effect of IFT88 deletion on GP ossification. (*A*) Micro‐CT partial 3D construction of AggrecanCreER^T2^;Ift88^fl/fl^ mice of naïve, off‐loaded, and contralateral joints. (*B*) Micro‐CT partial 3D construction of control and AggrecanCreER^T2^;Ift88^fl/fl^ wheel‐exercised joints. (*C*) Box plots (bars, maximum and minimum values; box is upper quartile and lower quartile with median) depict GP length of control and AggrecanCreER^T2^;Ift88^fl/fl^ mice in naïve, off‐loaded, contralateral, and wheel‐exercised mice. Statistical comparisons represent unpaired *t* tests, corrected for multiplicity, FDR 1%, ****p < 0.0001, *n* = 9–23.

In contrast to the initial hypothesis that primary cilia may be a positive regulator of the GP response to mechanical stress, we next tested if ciliary IFT88 could be regulating GP ossification, in a mechanically dependent manner, by protecting GP dynamics from disruptive mechanical force. *AggrecanCreER* ^*T2*^;*Ift88* ^*fl/fl*^ mice underwent double neurectomy surgery to off‐load the joint. In the vast majority of *AggrecanCreER* ^*T2*^;*Ift88* ^*fl/fl*^ mice, off‐loading inhibited the effects of IFT88 deletion on GP morphology (Fig. 4*A*); the difference in GP length between genotype was abolished by off‐loading (Fig. 4*C*). This effect was also evident in analysis of Safranin O‐stained histological sections (Supplemental Fig. S2*E*). The removal of ciliary IFT88 in conditions of increased mechanical loading (contralateral and wheel) did not influence the effects of increased loading on GP length.

Collectively, these data indicate that IFT88 is a mechanical force‐dependent regulator of the adolescent GP, ensuring coordinated ossification across the width of the limb. Next, we explored the cellular and molecular basis to these findings.

### Deletion of ciliary IFT88 inhibits cartilage resorption, “trapping” differentiated hypertrophic chondrocytes in expanded regions of the peripheral growth plate

**Fig. 5:**
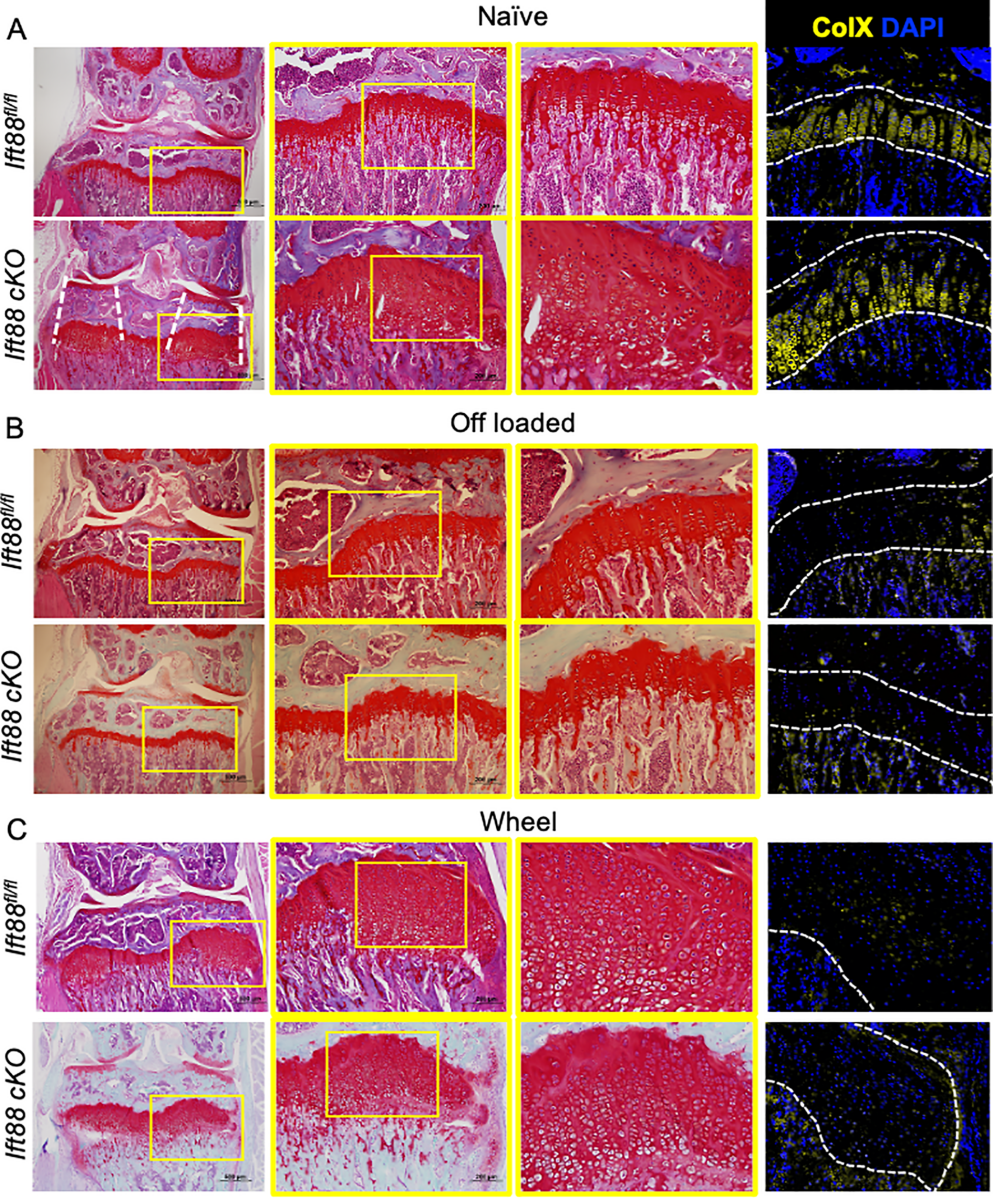
Impaired GP dynamics with IFT88 deletion and increased limb loading are associated with peripheral cartilaginous regions filled with disorganized hypertrophic chondrocytes. (*A*) Safranin O‐stained knee joints of naïve joints. Yellow boxes on 4× (left) and 10× (middle) images are enlarged to show GP (scale bar = 500 μm). White dotted lines highlight region of GP affected is directly beneath the articular surfaces. (*B*) Safranin O‐stained knee joints of off‐loaded joints. Yellow boxes on 4× (left) and 10× (middle) images are enlarged to show GP (scale bar = 500 μm). (*C*) Safranin O‐stained knee joints (4×) of wheel‐exercised joints. Yellow boxes on 4× (left) and 10× (middle) images are enlarged to show GP (scale bar = 500 μm). Representative images shown: *n* = 6–15 in all groups. AggrecanCreER^T2^;Ift88^fl/fl^ . (*A–C*) Analysis by immunohistochemistry to assess ColX protein expression. Counterstained with nuclear DAPI. White dashed lines outline the GP (*n* = 5 in all groups).

To understand the cellular and molecular mechanism underpinning the phenotype of *AggrecanCreER* ^*T2*^;*Ift88* ^*fl/fl*^ mice, we assessed the cellular and matrix composition of the peripheral regions of failed ossification by histology. Safranin O staining in naive *Ift88* ^*fl/fl*^ (control) animals revealed highly organized columns of chondrocytes in a small resting/proliferative population and larger hypertrophic population within the proteoglycan‐rich GP (Fig. 5*A*). In contrast in *AggrecanCreER* ^*T2*^;*Ift88* ^*fl/fl*^ animals, the disrupted peripheral regions directly beneath articular cartilage surfaces (Fig. 5*A*, dashed lines) were expanded regions of proteoglycan‐rich cartilage predominantly populated with swollen and disorganized, hypertrophic chondrocytes (Fig. 5*A*). In *Ift88* ^*fl/fl*^ (control) animals, collagen X staining (right‐hand panels of Fig. 5; IgG control shown in Supplemental Fig. S3*D*) was observed in the lower three‐quarters of the growth plate, likely co‐localized with prehypertrophic and hypertrophic chondrocyte populations. Collagen X staining was also present in the hypertrophic cells in *AggrecanCreER* ^*T2*^;*Ift88* ^*fl/fl*^ mice in both central (single arrow) and peripheral disrupted areas (double arrow). As the expanded regions of the growth plate were predominantly filled with cells surrounded by collagen X, the relative size of this area was increased in peripheral regions of *AggrecanCreER* ^*T2*^;*Ift88* ^*fl/fl*^ (Supplemental Fig. S3*A*). The intensity of staining was also increased in *AggrecanCreER* ^*T2*^;*Ift88* ^*fl/fl*^ (comparative quantitation shown in Supplemental Fig. S3*B*).

In contrast, the naïve context, off‐loaded limbs exhibited similar GP morphology in both *Ift88* ^*fl/fl*^ control and *AggrecanCreER* ^*T2*^;*Ift88* ^*fl/fl*^; however, comparatively to naïve mice, collagen X expression appeared reduced in both genotypes (Fig. 5*B*). However, in contralateral limbs, *Ift88* ^*fl/fl*^ control GP morphology was disrupted in a manner similar to naïve *AggrecanCreER* ^*T2*^;*Ift88* ^*fl/fl*^ mice (Supplemental Fig. S3*C*) with expanded peripheral regions filled with apparently hypertrophic chondrocytes. In contralateral limbs of *AggrecanCreER* ^*T2*^;*Ift88* ^*fl/fl*^, hypertrophic chondrocytes appeared even more enlarged. Wheel exercise also resulted in enlarged peripheral regions of cartilage full of disorganized hypertrophic chondrocytes, although, as observed in immobilized limbs, collagen X staining, while present and indeed enlarged due to peripheral disruption, signal was notably weaker (Supplemental Fig. S3*B*). This impaired ossification phenotype, observed in *AggrecanCreER* ^*T2*^;*Ift88* ^*fl/fl*^ mice during adolescence, is in stark contrast to that found with disruption of Hh signaling in embryonic and early postnatal mice, which results in accelerated hypertrophic differentiation and a reduced proliferation zone, resulting in premature ossification.^(^ ^29^, ^32^, ^33^ ^)^

Assessment of the relative populations of GP chondrocytes revealed no statistically significant changes in any population in *AggrecanCreER* ^*T2*^;*Ift88* ^*fl/fl*^ mice (Supplemental Fig. S3*E*) when compared with *Ift88* ^*fl/fl*^ control. The relative proportions of the populations were unchanged in all conditions when considering the middle of the growth plate. However, especially on the medial side, trends toward increases in the relative size of hypertrophic populations were observed with deletion of IFT88 and increased limb loading (wheel exercise). This indicated, in contrast to Hh disruption, a relative expansion of the hypertrophic populations. Indeed, non‐hypertrophic population sizes were not obviously affected. TUNEL staining revealed very low levels, and no differences, in apoptosis between control and *AggrecanCreER* ^*T2*^;*Ift88* ^*fl/fl*^ animals (Supplemental Fig. S3*F*), indicating the phenotype was not associated with an inhibition of cell death at the junction between GP cartilage and bone. Thus, in *AggrecanCreER* ^*T2*^;*Ift88* ^*fl/fl*^ mice, chondrocyte differentiation appeared uncoupled from GP ossification.

### Deletion of ciliary IFT88 is associated with increases in Hh signaling, which do not anatomically correlate to impairment of GP dynamics

To directly evaluate whether deletion of IFT88 altered GP hedgehog signaling, RNA*Scope* was performed to assess the expression of the Hh transcription factor *Gli1*, an indicator of pathway activity. *Gli1* expression was assessed on an individual cell basis and revealed that deletion of IFT88 was associated with small (13%) increases in *Gli1* expression as assessed by number of *Gli1*‐positive cells (Supplemental Fig. S4, ****p < 0.0001, *n* = 4 animals in each group). This increase was most predominant in the non‐hypertrophic chondrocytes (Supplemental Fig. S4*C*). No differences in *Gli1* expression were observed when comparing peripheral regions to central regions, suggesting changes to GP Hh signaling were not the primary cause of changes to GP dynamics in the peripheral regions upon deletion of IFT88.

### Deletion of ciliary IFT88 impairs osteoclastic recruitment to the peripheral growth plate

**Fig. 6:**
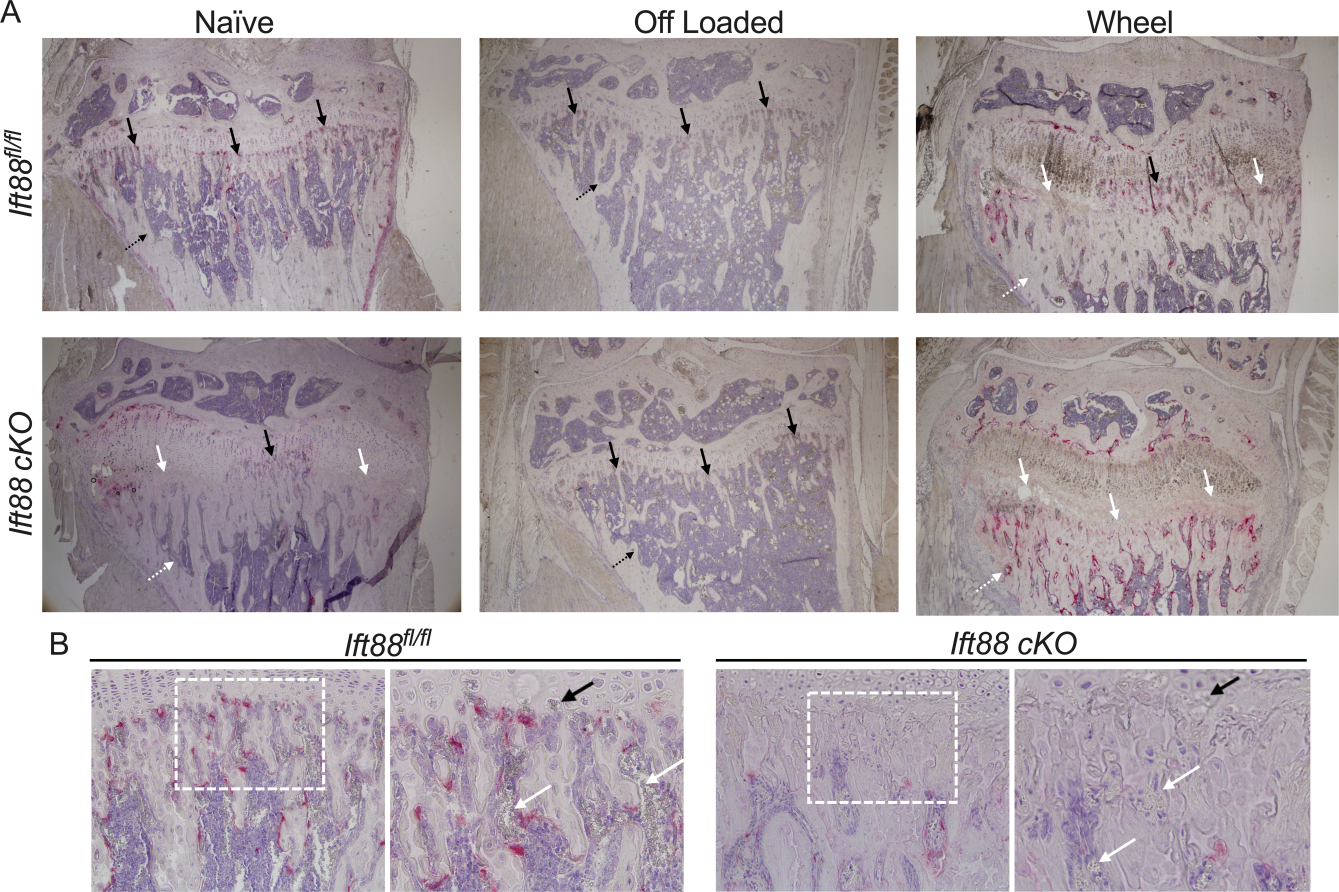
Wheel exercise and IFT88 deletion impairs osteoclast recruitment associated with failed ossification. (*A*) Representative TRAP and hemotoxylin staining in naïve, off‐loaded, and wheel joints. Black arrows point to normal osteoclastic activity in the primary spongiosa, whereas white arrows indicate where this staining is perturbed. Black dotted arrows point to normal trabecular bone, whereas white dotted arrows point to disrupted trabecular bone formation. (*n* = 5 in all groups). (*B*) Naïve control and AggrecanCreER^T2^;Ift88^fl/fl^ mice 20× images. White boxes show area zoomed in adjacent picture. White arrows show red blood cells in the bone marrow. Black arrows point to hypertrophic chondrocyte lacunae.

Enlarged growth plates are characteristic of protease knockout models.^(^ ^50^, ^51^ ^)^ It is still debated which cell types and proteases are responsible for GP resorption, but we first assessed chondroclastic and osteoclastic activity at the GP/bone frontier using tartrate‐resistant acid phosphatase (TRAP) staining. In naïve, *Ift88* ^*fl/fl*^ control mice, uniform clastic activity was observed along the chondro‐osseous junction (Fig. 6*A*, top left, black arrows). In contrast, in naive *AggrecanCreER* ^*T2*^;*Ift88* ^*fl/fl*^, TRAP staining was absent in peripheral regions of failed ossification (white arrows, Fig. 6*A*), whereas the central region was largely unaffected (black arrows). In off‐loaded *Ift88* ^*fl/fl*^ control joints, osteoclastic activity was reduced in the periosteum and trabeculae, but was still present along the chondro‐osseous junction across the width of the GP. In off‐loaded *AggrecanCreER* ^*T2*^;*Ift88* ^*fl/fl*^ mice, uniform osteoclastic activity was observed across the GP, thus rescuing differences between genotypes (Fig. 6*A*, top and bottom middle, black dotted arrows). Wheel exercise in control *Ift88* ^*fl/fl*^ mice resulted in similar osteoclastic activity to that observed in naïve *AggrecanCreER* ^*T2*^;*Ift88* ^*fl/fl*^ mice, namely a loss of TRAP staining in peripheral GP regions but not the central region (Fig. 6*A*, top and bottom right, black and white arrows). In exercised *AggrecanCreER* ^*T2*^;*Ift88* ^*fl/fl*^ mice, TRAP staining was absent from the chondro‐osseous junction across the width of the limb, but staining was more pronounced in the trabeculae below. Upon examination of the bone marrow at higher magnification, what appeared to be enucleated erythrocytes were visible in controls (Fig. 6*B*, left‐hand image, white arrows) at the chondro‐osseous frontier. These cells appeared to invade the remnant spaces left behind by a hypertrophic cell (Fig. 6*B*, left‐hand image, black arrow). Conversely, in *AggrecanCreER* ^*T2*^;*Ift88* ^*fl/fl*^ mice, there were far fewer erythrocytes and lack of bone marrow (Fig. 6*B*, right‐hand image, white arrows). Erythrocytes appeared unable to reach the growth plate to invade hypertrophic cell remnant shells (Fig. 6*B*, right‐hand image, black arrows).

### Deletion of ciliary IFT88 and altered limb mechanics disrupt growth plate cartilage VEGF expression and coordinated vascular invasion

**Fig. 7:**
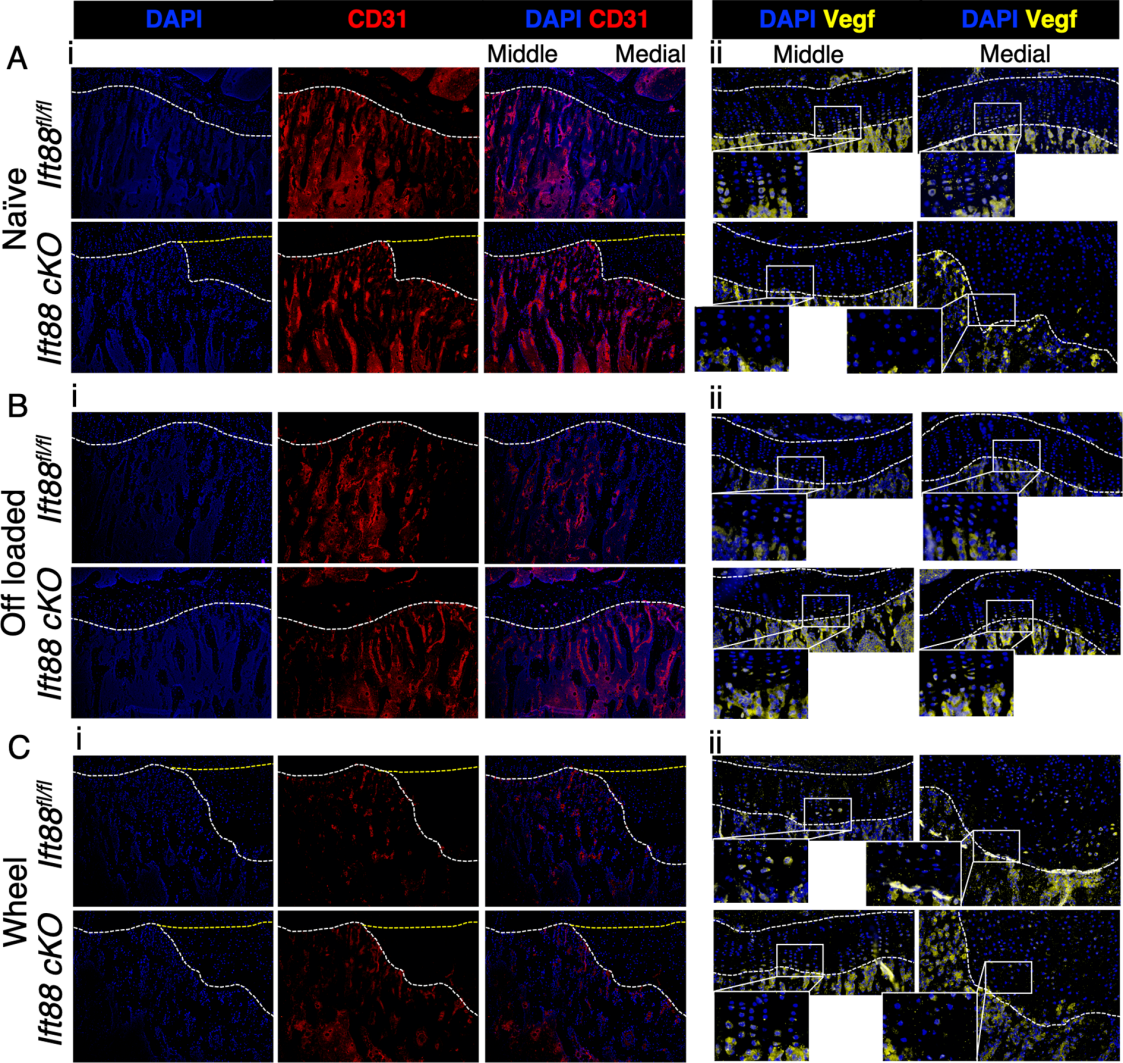
Ciliary IFT88 protects mechanosensitive expression of VEGF in hypertrophic chondrocytes. (*Ai*, *Bi*, *Ci*) Representative (10×) CD31staining (red) counterstained with nuclear stain DAPI (blue) in naïve (*Ai*), off‐loaded (*Bi*), and wheel‐exercised (*Ci*) Ift88^fl/fl^ control and AggrecanCreER^T2^;Ift88^fl/fl^ animals. White dashed lines demarcate the osteochondral junction between bone and GP cartilage. Yellow dashed lines indicate presumptive frontier of vascularization if not disrupted. (*Aii*, *Bii*, and *Cii*) Representative (20×) VEGF staining (yellow) counterstained with DAPI (blue) in naïve (*Aii*), off‐loaded (*Bii*), and wheel‐exercised (*Cii*) control and AggrecanCreER^T2^;Ift88^fl/fl^ animals. White dashed lines demarcate the GP. *n* = 3 for all groups, in both staining groups.

The carefully coordinated invasion of novel blood vessel types shapes limb development and has been shown to be critical to GP resorption during growth.^(^ ^52^ ^)^ Immunohistochemical staining of CD31, a blood vessel marker with particularly high expression in type H vessels in the metaphysis,^(^ ^53^ ^)^ revealed homogenous expression throughout the bone up to the osteochondral junction in *Ift88* ^*fl/fl*^ control animals (Fig. 7*Ai*, top panels). In contrast, peripheral regions of the GP cartilage where ossification failed in *AggrecanCreER* ^*T2*^;*Ift88* ^*fl/fl*^ mice, revealed vessels were absent in these areas (Fig. 7*Ai*, bottom panels). Off‐loading the joints restored vessel invasion in *AggrecanCreER* ^*T2*^;*Ift88* ^*fl/fl*^ with CD31 expression consistent across the width of the tibia in both genotypes (Fig. 7*Bi*). However, in both *Ift88* ^*fl/fl*^ control and *AggrecanCreER* ^*T2*^;*Ift88* ^*fl/fl*^ wheel‐exercised mice, failed regions of ossification were again associated with inhibited vascular recruitment in peripheral regions (Fig. 7*Ci*).

To coordinate vessel invasion of the epiphyseal cartilage, vascular endothelial growth factor (VEGF) is released by bone cells and hypertrophic chondrocytes. The genetic deletion of VEGF is also associated with enlarged GP.^(^ ^54^ ^)^ Histological sections were assessed for VEGF expression by IHC, revealing expression of VEGF in hypertrophic chondrocytes closest to bone in 10‐week‐old naïve *Ift88* ^*fl/fl*^ control mice and very strong staining in the primary spongiosa below (Fig. 7*Aii*, top panels). In contrast, *AggrecanCreER* ^*T2*^;*Ift88* ^*fl/fl*^ mice expressed little VEGF in regions of failed ossification and very low expression in the middle regions of the joint (Fig. 7*Aii*, bottom panels; quantification of signal in disrupted [medial] regions shown in Supplemental Fig. S5). VEGF expression across the GP/bone frontier was present in off‐loaded *AggrecanCreER* ^*T2*^;*Ift88* ^*fl/fl*^ mice; thus, immobilization restored uniform VEGF expression across the width of the limb at the chondro‐osseous junction. No robust changes in relative VEGF expression were observed with wheel exercise in either control or *AggrecanCreER* ^*T2*^;*Ift88* ^*fl/fl*^ mice, but localization of expression within the GP and bone beneath was disrupted (Fig. 7*Cii*). The unaffected central regions of the growth plate expressed VEGF in a tighter localization at the osteochondral junction.

Collectively, these data indicate ciliary IFT88 regulates the mechanosensitive expression of VEGF, ensuring coordinated invasion of blood vessels, osteoclastic recruitment, GP cartilage resorption, and ossification as skeletal growth draws to a close.

## Discussion

Research has repeatedly associated primary cilia with the cellular response to mechanical force, perhaps most famously in the context of propagating the kidney epithelial cell response to flow, perturbed in polycystic kidney disease.^(^ ^2^ ^)^ Given the congenital nature of the ciliopathies, the focus of cilia research has largely been on the cell and tissue development.^(^ ^55^ ^)^ Thus, our understanding of the roles of cilia in postnatal tissue has remained comparatively limited. We hypothesized that cilia, putative mechanotransduction organelles and established regulators of growth factor signaling, would maintain influence in the juvenile and adolescent limb, where pivotal tissue adaptations follow largely preprogrammed genetic instructions but are shaped by gradients of growth factor signaling and mechanical force. We have recently shown that ciliary *Ift88* is critical to the juvenile maturation and adult homeostasis of articular cartilage, safeguarding a program of calcification as cartilage matures.^(^ ^24^ ^)^ Here, we describe the effects of inducible and tissue‐specific deletion of ciliary *Ift88* in the progressive mineralization and ossification of the adolescent growth plate. We suggest the phenotype reveals how the potentially disruptive effects of mechanical forces are mitigated during this pivotal period in the limb, potentially illustrating an example of negative regulation of the response to mechanical force by the primary cilium.

Analysis of a reporter line revealed a mosaic activity of the *AggrecanCreER* ^*T2*^ used to delete *Ift88*. Given the localization of the phenotype in the *ift88*cKO model (*AggrecanCreER* ^*T2*^;*Ift88* ^*fl/fl*^), it's important to note no bias was observed to Cre activity between the center and periphery of the GP. By using *Ift88* ^*fl/fl*^ as our control, we controlled for any potential effects of tamoxifen, albeit our tamoxifen doses were below those characterized to effect bone structure.^(^ ^56^ ^)^ As we assessed tomato signal 2 weeks after tamoxifen administration, observed GP activity may be an underestimate of activity because of aggrecan‐positive lineages transdifferentiating to the primary spongiosa below. Nevertheless, Cre activity was apparent in resting, potentially recycling, populations at the top of the growth plate and columns beneath. Potentially, therefore, the Cre is active in recently described stem cell populations.^(^ ^25^, ^57^ ^)^ Although the slowing of supply of new cells to the GP may well underpin epiphyseal senescence in adolescence,^(^ ^26^ ^)^ the progression of chondrocyte lineages within *ift88*cKO GP does not suggest stem cell renewal or differentiation has been affected but rather chondrocyte hypertrophy and GP exit is inhibited. The observation of tomato‐positive cells in bone is suggestive of translocation from GP to bone at this time point but does also raise the prospect that bone‐resident populations have been directly affected by the Aggrecan Cre. Roles for cilia in bone progenitors^(^ ^58^ ^)^ and osteocyte biology and mechanobiology^(^ ^4^ ^)^ have been previously described; thus, changes to the bone architecture beneath the GP may be: (i) the direct result of Cre activity on bone cell populations; (ii) indirect effects due to altered limb biomechanics or alterations in upstream ossification; and/or (iii) the result of an impact on chondrocyte transdifferentiation into bone. In turn, it is possible that changes to this architecture impede vessel invasion and thus the continued ossification of the growth plate. As such, we absolutely would not rule out cell‐extrinsic factors and chondrocyte‐independent mechanisms underlying the phenotypes observed.

Our IHC analysis was able to confirm reductions in cilia number in the GP. We cannot rule out that deletion of *Ift88* might have non‐ciliary effects, including through changes to the cytoskeleton. Previous models targeting *Ift88* have documented changes to the chondrocyte cytoskeleton, implicated in regulating cellular strain,^(^ ^59^ ^)^ and more specifically in F‐actin networks leading to failed hypertrophic reprogramming of chondrocytes.^(^ ^22^ ^)^ Furthermore, IFT proteins including IFT88 have recently been shown to interact directly with the Hippo effector YAP1 in a ciliary‐independent manner.^(^ ^60^ ^)^ Cartilage‐specific disruption of YAP‐TAZ also results in altered limb morphogenesis^(^ ^61^ ^)^ but associated with changes in extracellular matrix.

The effects of the conditional, inducible deletion of *Ift88* we describe here contrast with that found in the GP of Hh and cilia models earlier in development,^19^, ^29^, ^62^ ^)^ where Hh is a pro‐proliferative signal, which, when lost, results in premature GP ossification and complete closure. This immediately suggested that either the roles of Hh are altered in the adolescent GP or the most important role of cilia in the GP, at this age, is not the tuning of an Hh signal but regulation of the cell and tissue response to another external cue. Our RNAscope analyses suggest ciliary IFT88 may be a negative regulator of Hh signaling but throughout the width of the GP.

In contrast to the homogenous, relatively subtle changes to Hh, the appearance of regions of failed ossification, directly beneath the load‐bearing articular cartilage plateaus, implicated an anatomical heterogeneity of tissue remodeling, potentially downstream to anisotropic tissue mechanics across the width of the limb.^(^ ^46^, ^47^ ^)^ This led us to hypothesize that the loss of cilia was altering GP sensitivity to mechanical force, with ramifications in peripheral regions of the tibial GP that modeling suggests experience greater stresses.^(^ ^46^, ^47^ ^)^ Thus, our interpretation is that the primary underlying mechanism in *ift88*cKO adolescent growth plates is altered mechanoadaptation, potentially independently of the cilium's role in tuning Hh signaling and at least in part through disruption of VEGF at the ossification front.

Previous studies have investigated the effects of changes to mechanical loading during growth. Harnessing an extra 10% body weight to chickens^(^ ^63^ ^)^ resulted in narrowing of the GP and enhancements of ossification and vascularization. In contrast, the mimicry of high‐impact exercise in juvenile rats monitored from 4 to 12 weeks of age also limited growth but was associated with increased GP length.^(^ ^64^ ^)^ We were surprised to find that simply the increased compensatory loading in the contralateral limb of our double neurectomy experiments or the provision of a wheel for exercise for 2 weeks resulted in striking inhibition of peripheral ossification of the GP in control mice, which very closely resembled that found with conditional deletion of IFT88. We would assume these rapid effects might recover with time through a reactive feedback adaptation of morphogenesis. We propose they represent an acute change for relatively sedentary caged mice, but they nevertheless demonstrate the sensitivity of GP dynamics at this time point. Longer‐term or longitudinal studies would be required to explore this. The results extend the rationale for more research into the effects of acute changes to biomechanics during adolescence. In addition to modeling the effects of loading in a number of animal models, cross‐sectional studies of adolescents engaged in physical activity demonstrate that sporting activity is strongly associated with epiphyseal extension and hypertrophy and the development of CAM morphology, itself a strong risk factor for hip pain and the development of osteoarthritis (OA) in humans.^(^ ^65^ ^)^ A better understanding of the interactions between mechanical loading and the maturing skeleton will not only strengthen our appreciation of the risks associated and pathological processes that underlie common pathologies such as OA, but also other mechanically associated chondropathies.

We propose that cilia might act to dampen or threshold the cellular response to mechanical forces in the GP that might otherwise be disruptive to its coordinated ossification, predisposing the limb to poor health later in life. Cilia have been proposed to play a critical role in mechanotransduction in chondrocytes and/or the GP before, on the basis of both correlations between loading and cilia prevalence in situ^(^ ^48^ ^)^ and in vitro evidence from chondrocyte cell lines.^(^ ^6^, ^48^, ^66^ ^)^ Cilia have recently been shown to play a critical role integrating mechanical loading and force in tendon.^(^ ^67^ ^)^ Furthermore, the removal of cilia in the vascular endothelium left turbulent regions of the vasculature predisposed to the formation of atherosclerotic plaques,^(^ ^68^ ^)^ perhaps another example of “mechanoflammation” recently coined in the OA field.^(^ ^69^ ^)^ We have previously investigated apparent roles for ciliary proteins in the cellular response to inflammatory cues.^(^ ^70^, ^71^ ^)^ It has also been proposed that primary cilia sensitize endothelial cells to bone morphogenetic proteins to regulate vessel morphogenesis in low‐flow conditions.^(^ ^72^ ^)^

**Fig. 8:**
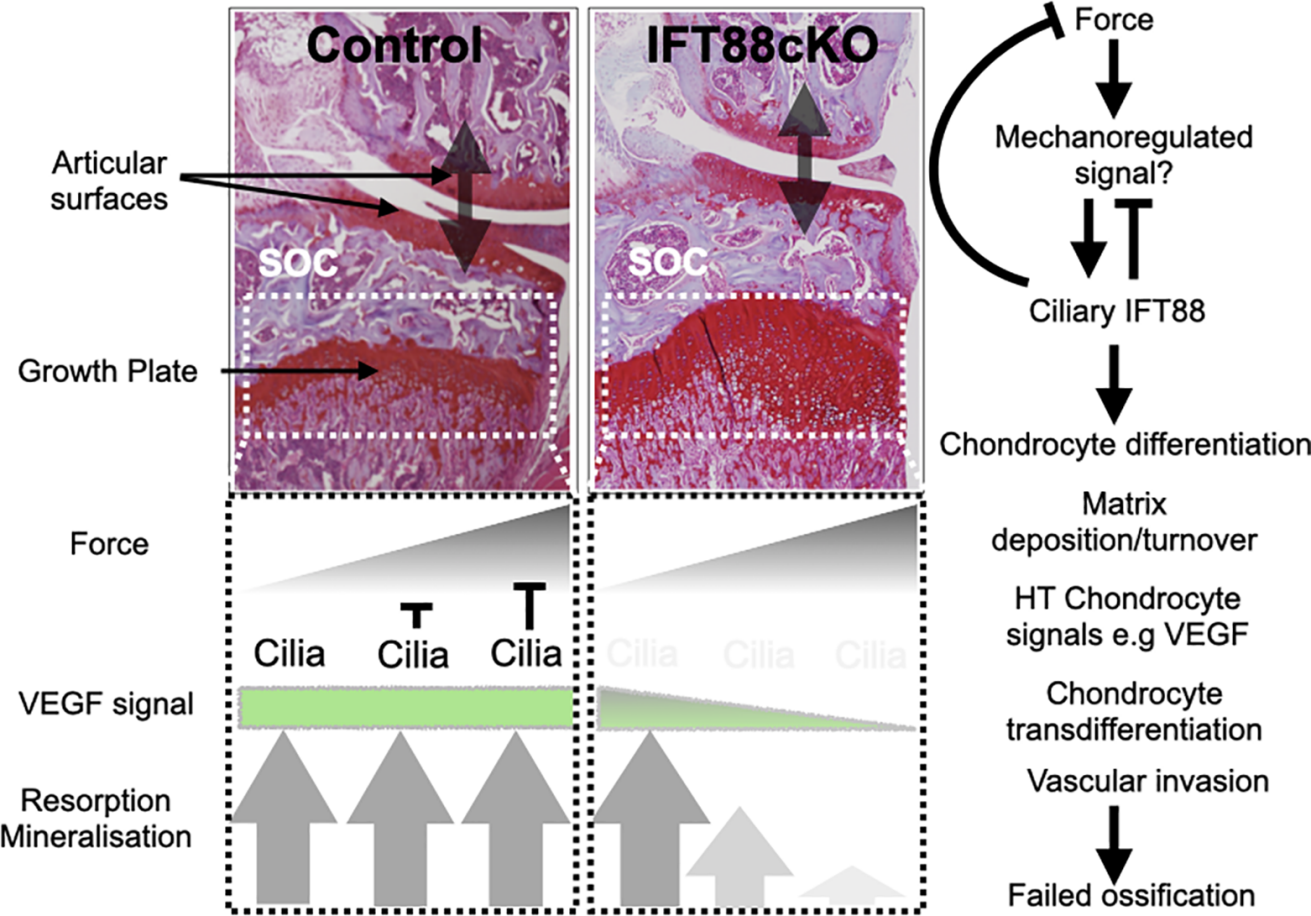
Proposed role of cilia in ensuring coordinated GP dynamics in face of disruptive force heterogeneity across the limb. As modeled previously, in control (normal) scenario, a gradient of force exists across the width of limb, as a result of load bearing at the articular surfaces and imperfect redistribution of stresses by secondary ossification (SOC) center. To ensure the rate of mineralization, vascular invasion, cartilage resorption, and ossification is uniform across the width of the growth plate, cilia support even expression of VEGF by hypertrophic chondrocytes at osteochondral frontier. We propose this “dampening” of response to force, either directly or indirectly through a yet to be identified mechanoregulated signal, to higher stresses at the periphery, and/or potentially sensitization of chondrocytes in central regions experiencing lower stresses, is lost with depletion of cilia (IFT88cKO). Potentially as a result of altered hypertrophic differentiation and/or changes to the extracellular matrix, the uniform VEGF signal is disrupted, resulting in uneven advance in the peripheral regions associated with “trapping” of hypertrophic chondrocytes. Histology images are both medial regions of joints from Ift88^fl/fl^ (left) and AggrecanCreER^T2^;Ift88^fl/fl^ (right), respectively.

As such, cilia appear to act at the interface between biological and biophysical programs, with very likely a cell type and tissue niche specificity. Our interpretation of the in vivo studies presented here is that in the adolescent epiphysis, in the absence of the influence of cilia, the differential of force, and thereby likely cellular strain in the hypertrophic region, across the width of the GP, leads to a heterogeneity of VEGF expression, a disrupted rather than tightly controlled expression pattern at the chondro‐osseous junction (Fig. 8). This may be the result of an altered program of differentiation, due to changes in collagen X deposition (present in *ift88* cKO but diminished in immobilized and wheel‐exercise conditions) or other matrix components. Matrix turnover is clearly affected, not least indicated by the failure of cartilage resorption. Collectively, these changes may result in disruption to VEGF, chondrocyte transdifferentiation, vascular invasion, and ultimately the mineralization and ossification of the growth plate in these regions.

As demonstrated previously in vitro,^(^ ^73^, ^74^ ^)^ limb VEGF expression is mechanosensitive in situ, as indicated by its scattered nature in wheel‐exercised mice and the reductions found in the bone with immobilization. *AggrecanCreER* ^*T2*^;*Ift88* ^*fl/fl*^ mice exhibited a loss of VEGF expression that we propose impairs the recruitment of type H vessels^(^ ^52^ ^)^ and osteoclasts and inhibits cartilage resorption, hypertrophic chondrocyte transdifferentiation, and ultimately the coordinated ossification of the GP. These effects were all inhibited upon off‐loading the limb by immobilization, demonstrating the requirement of physiological loading for the *ift88* cKO phenotype. Liu and colleagues^(^ ^75^ ^)^ demonstrated that deletion of the retrograde ciliary IFT80 using a Col2a1 Cre to constitutively delete in chondrocytes impaired chondrocyte differentiation in the context of fracture healing. The authors reported reduced angiogenesis and VEGF mRNA expression in this context. VEGF function in vascularization has also recently been linked to cilia in pancreatic islets,^(76^ ^)^ albeit in the context of ligand internalization and downstream signaling rather than expression. Although VEGF expression is mechanoregulated, the nature of the mechanical stress and/or identity of transducing signals upstream to VEGF expression, that IFT88 dampens the response to, remains an open question. A holistic characterization of changes to cellular phenotype, matrix composition, and turnover and cellular signaling in the regions disrupted by deletion of IFT88 or changes to mechanical loading will help identify more about both the processes that IFT88 regulates and the mechanoregulation of growth plate ossification.

Our results describing IFT88 depletion in the growth plate and in the articular cartilage^(^ ^24^ ^)^ suggest it is critical to safeguarding adolescent chondrocyte phenotypic programs in both cartilage types, as has been recently described for SOX9.^(^ ^77^ ^)^ Both adaptability and resilience to mechanical forces are critical to tissue maturation and health. We conclude that ciliary Ift88 plays a critical role in this context in the juvenile and adolescent growth plate, its removal resulting in failed resorption and ossification of the growth plate at the end of growth. This phenomenon, observed in *AggrecanCreER* ^*T2*^;*Ift88* ^*fl/fl*^ mice, is dependent on mechanical force, implying that IFT88, and potentially by extension the primary cilium, acts as a “mechano‐dampener,” protecting carefully coordinated epiphyseal biology from otherwise disruptive mechanics.

## Disclosures

TLV served as an ad hoc consultant in the past 3 years for Mundipharma and GSK. All other authors state that they have no conflicts of interest.

## Author Contributions

**Clarissa Rosalind Coveney:** Conceptualization; data curation; formal analysis; investigation; methodology; writing – review and editing. **Hasmik Jasmine Samvelyan:** Formal analysis; investigation; methodology; visualization; writing – review and editing. **Jadwiga Miotla‐Zarebska:** Investigation; methodology; project administration; resources; writing – original draft. **Josephine Carnegie:** Data curation; formal analysis; investigation; methodology. **Emer Chang:** Formal analysis; investigation. **Jonty Corrin:** Formal analysis; investigation. **Trystan Coveney:** Formal analysis; investigation. **Bryony Stott:** Data curation; formal analysis; investigation; methodology; project administration; resources. **Ida Parisi:** Data curation; formal analysis; investigation; methodology; project administration; resources. **Claudia Duarte:** Investigation; methodology; project administration; resources. **Tonia Vincent:** Conceptualization; funding acquisition; methodology; resources; supervision; writing – review and editing. **Katherine Staines:** Conceptualization; formal analysis; investigation; resources; supervision; writing – original draft; writing – review and editing. **Angus Wann:** Conceptualization; data curation; formal analysis; funding acquisition; investigation; methodology; project administration; resources; supervision; visualization; writing – original draft; writing – review and editing.

### Peer Review

The peer review history for this article is available at https://publons.com/publon/10.1002/jbmr.4502.

## Supporting information


**Supplemental Fig. S1.** (*A*) IHC staining for primary cilia in GP tissue sections from control and AggrecanCreERT2;Ift88fl/fl animals. Scale bar = 20 μM. White arrows indicate clearly identifiable primary cilia positive for both Acetylated‐α‐tubulin (green) and ARL13B (magenta). DAPI staining (blue) indicates nuclei. (*B*) Cilia‐positive and cilia‐negative counts taken from 6 regions of growth plate across tibia from *n* = 4 mice. Fisher's exact test shown ****p < 0.0001. (*C*) Eight points of GP length measurements (yellow lines) across representative single micro‐CT section. (*D*) Box plot (bars, maximum and minimum values, box is upper quartile and lower quartile with median) depicts GP length as measured from Safranin O‐stained histological sections. Analyzed by one‐way ANOVA, *p < 0.05, **p < 0.01, *n* = 9–12.
**Supplemental Fig. S2.** (*A*) Partial 3D reconstruction of μCT scan to show the 15 primary spongiosa region of bone directly below GP analyzed and associated BV/TV 16 (%) quantitation. (*B*) Tibia width measurements (from μCT). Pairwise unpaired *t* tests, corrected for multiplicity shown. *p = <0.05. (*C*) GP length measurements from Safranin O histological sections (lateral, central, and medial regions, left to right, as quantified in box plots in (*D*). (*E*) Box plots (bars, maximum and minimum values, box is upper quartile and lower quartile with median) depict GP length of control and AggrecanCreERT2;Ift88fl/fl mice in naïve, off‐loaded, contralateral, and wheel‐exercised mice, *n* = 9–23.
**Supplemental Fig. S3.** Box plots (bars, maximum and minimum values, box is upper quartile and lower quartile with median) depict area (*A*) and intensity per unit area (*B*) of Collagen X staining (*n* = 5 in each group). Signal quantified across full width of GP. (*C*) Safranin O‐stained knee joints of contralateral joints from control and AggrecanCreERT2;Ift88fl/fl animals. Yellow boxes on 4× (left) and 10× (middle) images are enlarged to show GP (scale bar = 500 μm). (*D*) Rabbit IgG control for conditions matched to Collagen X staining. (*E*) Analysis of relative (%) hypertrophic and non‐hypertrophic GP chondrocyte populations. Two‐way ANOVA with multiple comparison tests shown (*n* = 6‐15). (*F*) TUNEL staining (green) in control and AggrecanCreERT2;Ift88fl/fl animals. White arrows highlight TUNEL‐positive cells in GP.
**Supplemental Fig. S4.** (*A*) Representative RNAScope of Gli1 expression in GP on the medial side of control and AggrecanCreERT2;Ift88fl/fl animals, counterstained with 41 DAPI (blue) (*n* = 4 in each group). White dashed lines demarcate GP. White dashed box shows enlarged regions in adjacent image. (*B*) Contingency data of Gli1‐positive nuclei (analyzed by Fisher's exact test, ****p < 0.0001, % Gli1‐positive shown in white) in naïve control and AggrecanCreERT2;Ift88fl/fl mice (*n* = 4 minimum in all groups). (*C*) Contingency data of Gli1‐positive nuclei in non‐hypertrophic and hypertrophic regions of the GP to assess Gli1 expression by cell positivity (analyzed by Fisher's exact test, **p < 0.01) in naïve and AggrecanCreERT2;Ift88fl/fl mice (*n* = 4 in all groups).
**Supplemental Fig. S5.** Violin plots quantifying VEGF expression assessed by IHC (Fig. 7). Statistical comparisons are Fisher's exact test, *n* = 3 in all groups.

## Data Availability

Data available on request from the authors
