# Supplementary material for: Ciliary IFT88 Protects Coordinated Adolescent Growth Plate Ossification From Disruptive Physiological Mechanical Forces
Source: J Bone Miner Res. 2022 Feb 20;37(6):1081–96. doi: 10.1002/jbmr.4502 (PMC9304194; doi:10.1002/jbmr.4502)
Supplement: Supplementary file 1 — Supplemental Fig. S1. (A) IHC staining for primary cilia in GP tissue sections from control and AggrecanCreERT2;Ift88fl/fl animals. Scale bar = 20 μM. White arrows indicate clearly identifiable primary cilia positive for both Acetylated‐α‐tubulin (green) and ARL13B (magenta). DAPI staining (blue) indicates nuclei. (B) Cilia‐positive and cilia‐negative counts taken from 6 regions of growth plate across tibia from n = 4 mice. Fisher's exact test shown ****p < 0.0001. (C) Eight points of GP length measurements (yellow lines) across representative single micro‐CT section. (D) Box plot (bars, maximum and minimum values, box is upper quartile and lower quartile with median) depicts GP length as measured from Safranin O‐stained histological sections. Analyzed by one‐way ANOVA, *p < 0.05, **p < 0.01, n = 9–12. Supplemental Fig. S2. (A) Partial 3D reconstruction of μCT scan to show the 15 primary spongiosa region of bone directly below GP analyzed and associated BV/TV 16 (%) quantitation. (B) Tibia width measurements (from μCT). Pairwise unpaired t tests, corrected for multiplicity shown. *p = <0.05. (C) GP length measurements from Safranin O histological sections (lateral, central, and medial regions, left to right, as quantified in box plots in (D). (E) Box plots (bars, maximum and minimum values, box is upper quartile and lower quartile with median) depict GP length of control and AggrecanCreERT2;Ift88fl/fl mice in naïve, off‐loaded, contralateral, and wheel‐exercised mice, n = 9–23. Supplemental Fig. S3. Box plots (bars, maximum and minimum values, box is upper quartile and lower quartile with median) depict area (A) and intensity per unit area (B) of Collagen X staining (n = 5 in each group). Signal quantified across full width of GP. (C) Safranin O‐stained knee joints of contralateral joints from control and AggrecanCreERT2;Ift88fl/fl animals. Yellow boxes on 4× (left) and 10× (middle) images are enlarged to show GP (scale bar = 500 μm). (D) Rabbit IgG control for cond [file JBMR-37-1081-s001.docx]

**Supplementary Data.**


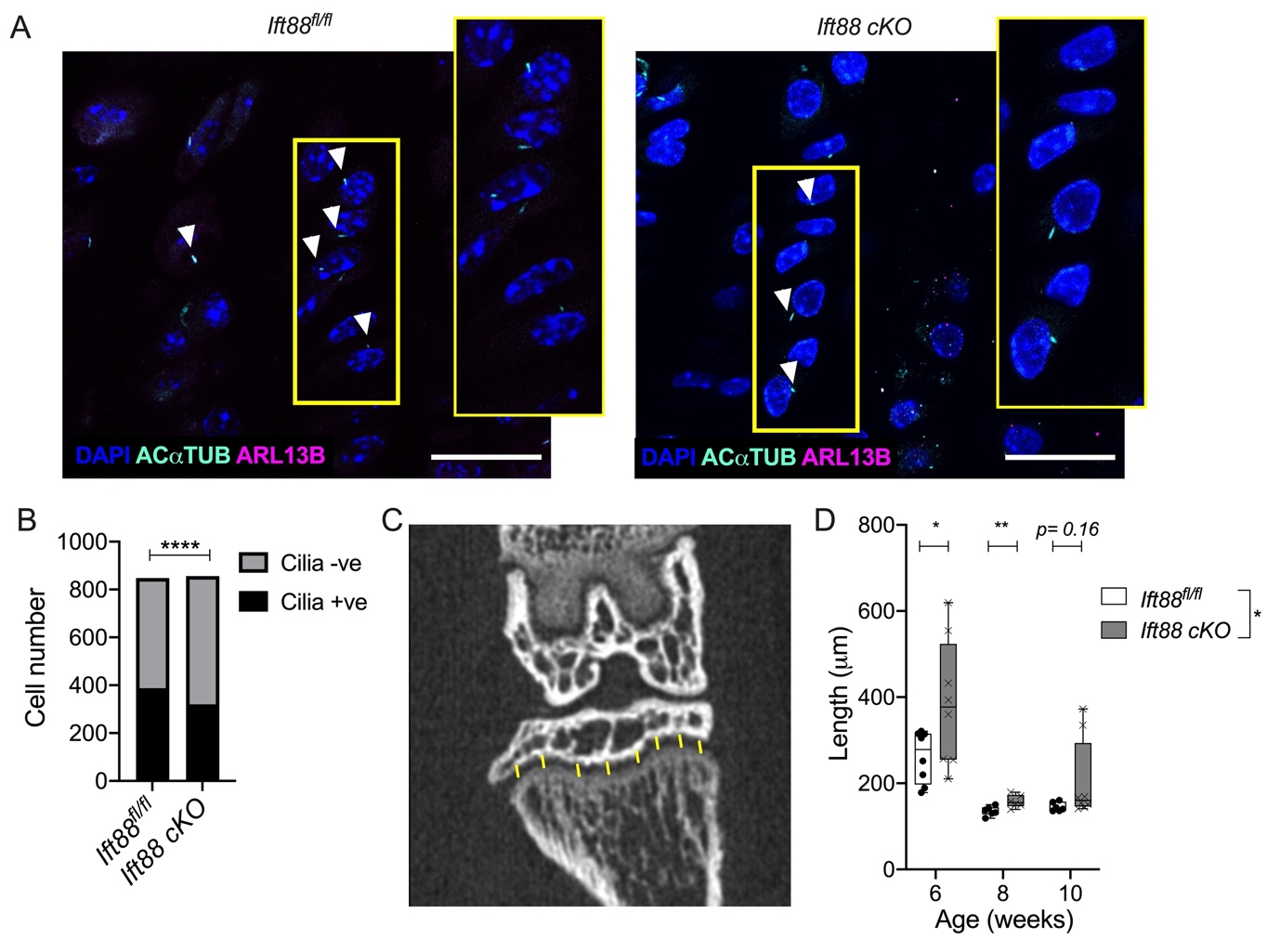


***Supplementary Figure 1. A,*** *IHC staining for primary cilia in GP tissue sections from control and AggrecanCreER^T2^;Ift88^fl/fl^ animals. Scale bar 20μM. White arrows indicate clearly identifiable primary cilia positive for both Acetylated-α-tubulin (green) and ARL13B (magenta). DAPI staining (blue) indicates nuclei.* ***B*** *Cilia positive and cilia negative counts taken from 6 regions of growth plate across tibia from n=4 mice. Fisher’s exact test shown ****p<0.0001.* ***C*** *8 points of GP length measurements (yellow lines) across representative single MicroCT section.* ***D*** *Box plot (Bars, maximum and minimum values, box is upper quartile and lower quartile with median) depicts GP length as measured from Safranin-O stained histological sections. Analysed by one-way ANOVA, *p<0.05, **p<0.01 n=9-12****.***


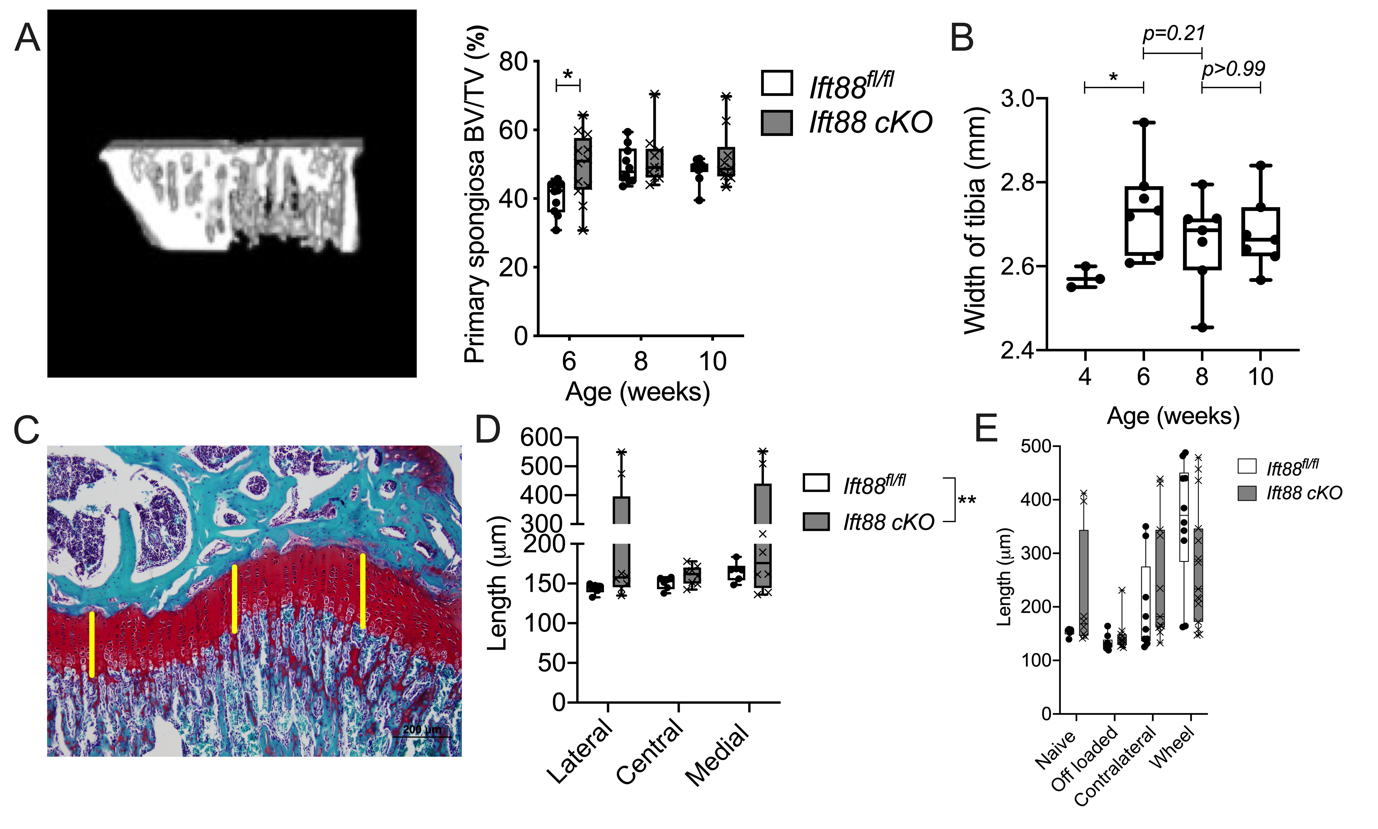


***Supplementary Figure 2. A,*** *Partial 3D reconstruction of uCT scan to show the primary spongiosa region of bone directly below GP analysed and associated BV/TV (%) quantitation.* ***B*** *Tibia width measurements (from uCT). Pairwise unpaired-t-tests, corrected for multiplicity shown. *p=<0.05.* ***C*** *GP length measurements from Safranin-O histological sections (lateral, central and medial regions, left to right, as quantified in box plots in* ***D****.* ***E.*** *Box plots (Bars, maximum and minimum values, box is upper quartile and lower quartile with median) depict* *GP length of control and AggrecanCreER^T2^;Ift88^fl/fl^ mice in naïve, off-loaded, contralateral and wheel exercised mice, n=9-23.*


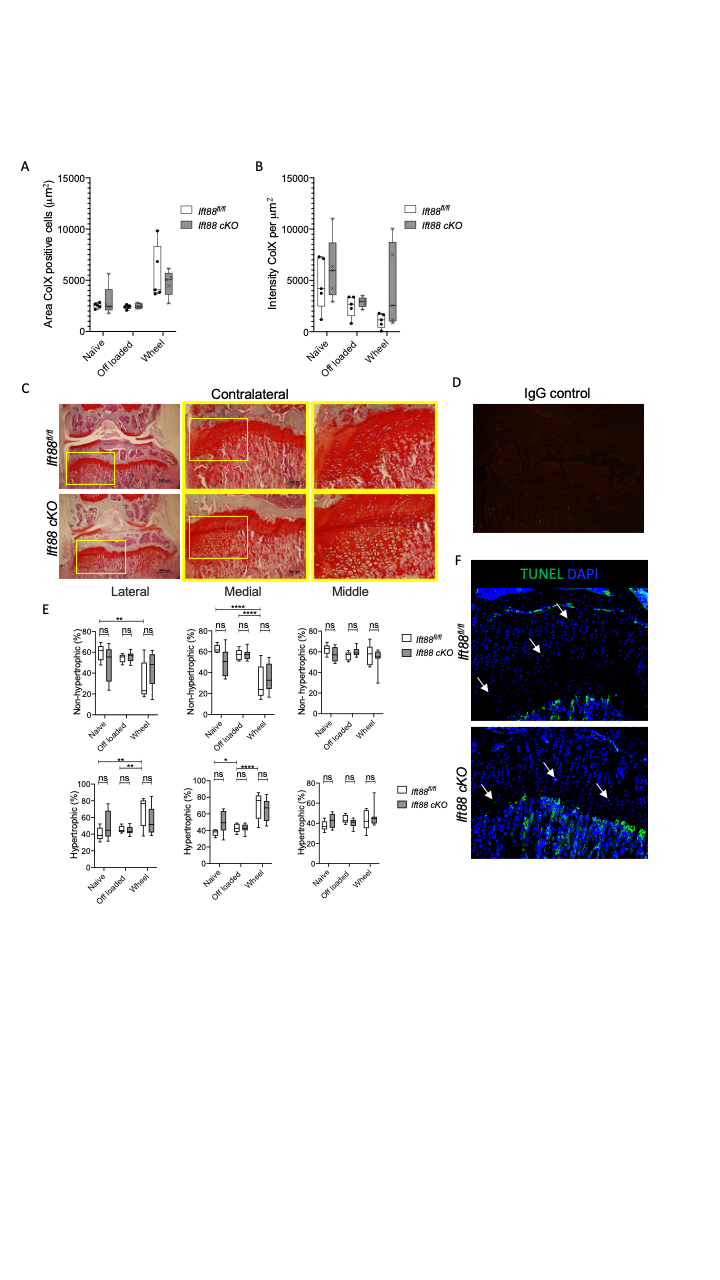


***Supplementary Figure 3. A/B*** *Box plots (Bars, maximum and minimum values, box is upper quartile and lower quartile with median) depict area (A) and intensity per unit area (B) of Collagen X staining (n=5 in each group). Signal quantified across full width of GP.*  ***C*** *Safranin O stained knee joints* *of contralateral joints from control and AggrecanCreER^T2^;Ift88^fl/fl^ animals. Yellow boxes on 4x (left) and 10x (middle) images are enlarged to show GP, (scale bar= 500μm).****D,*** *Rabbit IgG* *control for conditions matched to Collagen X staining.* ***E*** *Analysis of relative (%) hypertrophic and non-hypertrophic GP chondrocyte populations. Two-way ANOVA with multiple comparison tests shown (n=6-15).* ***F*** *TUNEL staining (green) in control and AggrecanCreER^T2^;Ift88^fl/fl^ animals. White arrows highlight TUNEL positive cells in GP.*


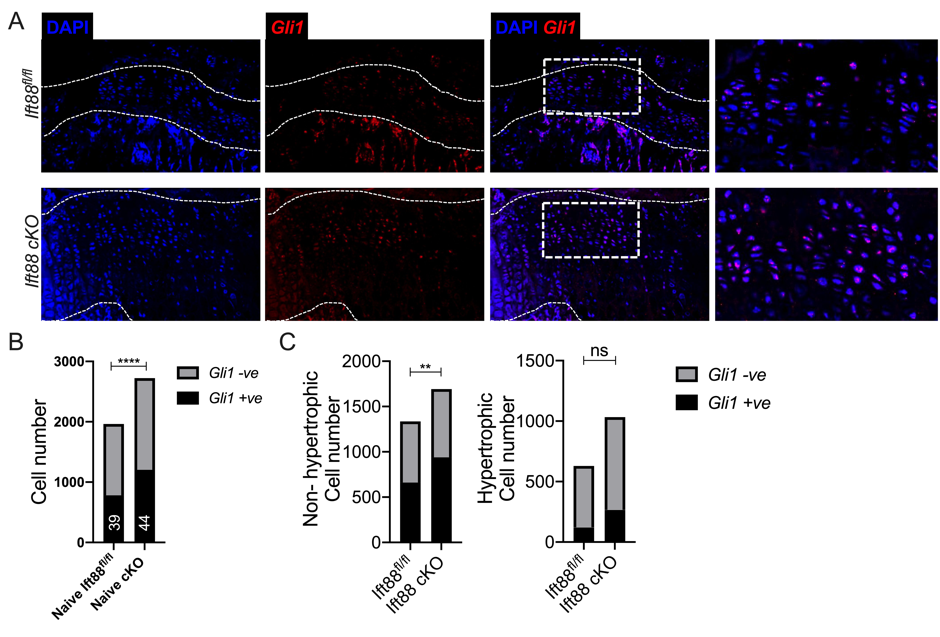


***Supplementary Figure 4. A,*** *Representative RNAScope of Gli1 expression in GP on the medial side of control and AggrecanCreER^T2^;Ift88^fl/fl^ animals, counterstained with DAPI (blue) (n=4 in each group). White dashed lines demarcate GP. White dashed box shows enlarged regions in adjacent image.* ***B,*** *Contingency data of Gli1 positive nuclei (Analysed by Fisher’s exact test, ****p<0.0001, % Gli1 positive shown in white) in naïve control and AggrecanCreER^T2^;Ift88^fl/fl^ mice (n= 4 minimum in all groups).* ***C,*** *Contingency data of Gli1 positive nuclei in non-hypertrophic and hypertrophic regions of the GP to assess Gli1 expression by cell positivity (Analysed by Fisher’s exact test, **p<0.01) in naïve and AggrecanCreER^T2^;Ift88^fl/fl^ mice (n= 4 in all groups).*


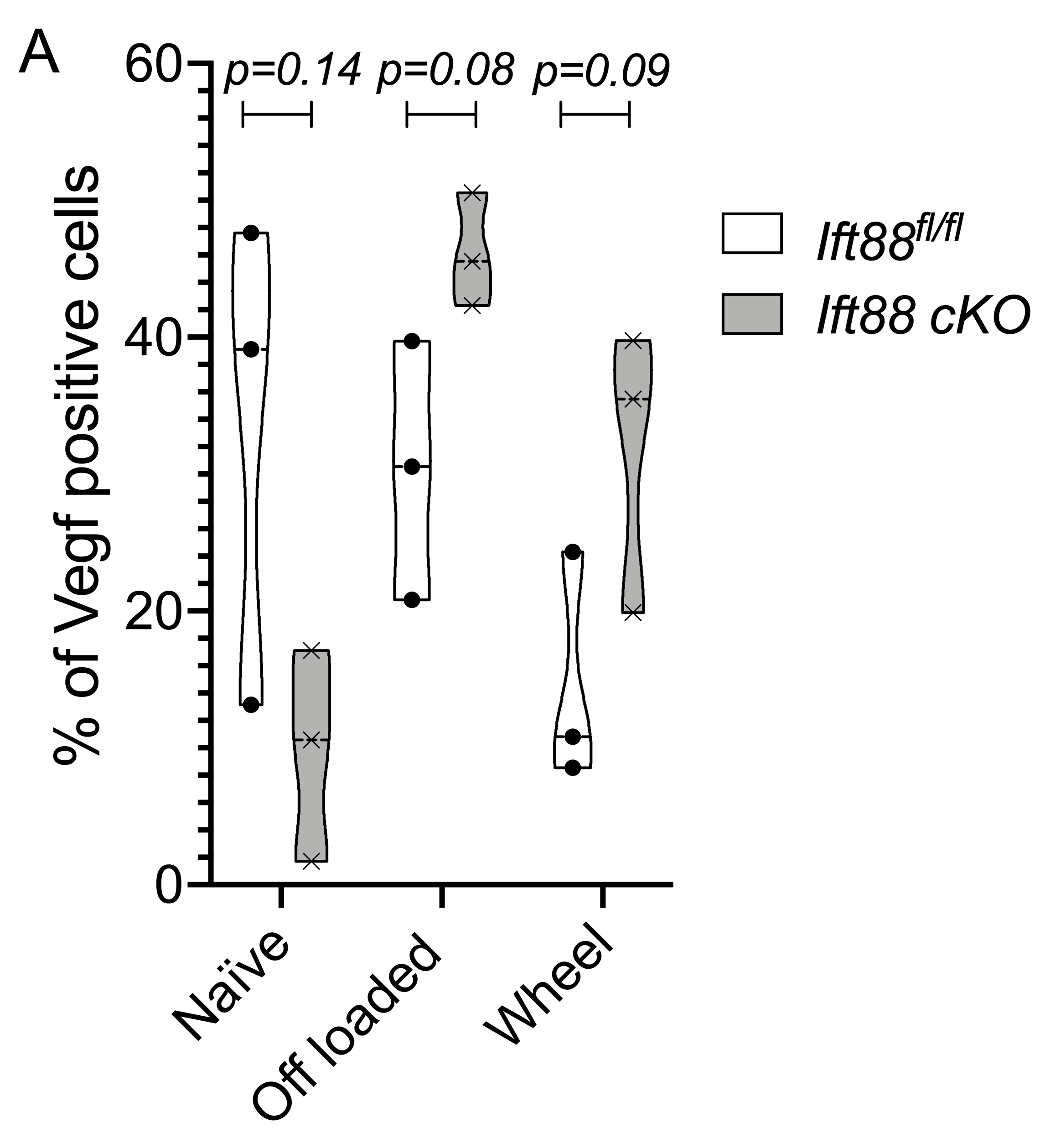


***Supplementary Figure 5.*** *Violin plots quantifying VEGF expression assessed by IHC (Figure 7). Statistical comparisons are Fisher’s exact test, n=3 in all groups.*
